# Targeting mitochondrial reactive oxygen species as novel therapy for inflammatory diseases and cancers

**DOI:** 10.1186/1756-8722-6-19

**Published:** 2013-02-25

**Authors:** Xinyuan Li, Pu Fang, Jietang Mai, Eric T Choi, Hong Wang, Xiao-feng Yang

**Affiliations:** 1 Cardiovascular Research Center, Department of Pharmacology and Thrombosis Research Center, Temple University School of Medicine, 3500 North Broad Street, Philadelphia, PA 19140, USA; 2 Cardiovascular Research Center and Department of Surgery, Temple University School of Medicine, 3500 North Broad Street, Philadelphia, PA 19140, USA

**Keywords:** Mitochondria, ROS, Inflammatory diseases

## Abstract

There are multiple sources of reactive oxygen species (ROS) in the cell. As a major site of ROS production, mitochondria have drawn considerable interest because it was recently discovered that mitochondrial ROS (mtROS) directly stimulate the production of proinflammatory cytokines and pathological conditions as diverse as malignancies, autoimmune diseases, and cardiovascular diseases all share common phenotype of increased mtROS production above basal levels. Several excellent reviews on this topic have been published, but ever-changing new discoveries mandated a more up-to-date and comprehensive review on this topic. Therefore, we update recent understanding of how mitochondria generate and regulate the production of mtROS and the function of mtROS both in physiological and pathological conditions. In addition, we describe newly developed methods to probe or scavenge mtROS and compare these methods in detail. Thorough understanding of this topic and the application of mtROS-targeting drugs in the research is significant towards development of better therapies to combat inflammatory diseases and inflammatory malignancies.

## Introduction

Free radicals and other ROS are generated in a wide range of normal physiological conditions. However, ROS also participate in many pathological conditions including cardiovascular diseases, malignancies, autoimmune diseases, and neurological degenerative diseases. Despite intensive investigations in this field, current anti-oxidant therapeutics are not clinically effective in combating these pathological conditions suggesting that our understanding of this field is limited, and there is a need to narrow the “knowledge gap” in order to develop more effective new therapies [1]. Although ROS are historically considered toxic by-products of cellular metabolism, recent studies have suggested that cells “have learned” to harness the power of ROS for cell signaling purposes. In analogous to phosphorylation modification of proteins, the term “redox signaling” is emerging in reference to events of oxidation modification of proteins by ROS. Indeed, there are multiple sources of ROS in the cell including nicotinamide adenine dinucleotide phosphate (NADPH) oxidase (NOX) [2], xanthine oxidase (XO), uncoupling of nitric oxide synthase (NOS), cytochrome P450, and mitochondrial electron transport chain (ETC). Among these potential sources, however, mtROS have drawn increasing attentions because it was recently discovered that mtROS directly contribute to inflammatory cytokine production and innate immune responses [3] by activation of newly characterized RIG-I-like receptors (RLRs) [4], inflammasomes [5], and mitogen-activated protein kinases (MAPK) [6].

Cardiovascular disease (CVD) is the leading cause of morbidity and mortality in the western world. Nearly 75% of the CVD-related death results from atherosclerosis which is found in 80-90% of Americans over the age of 30. Early atherosclerotic lesions can be detected in youths as young as 7 years of age [7,8]. As a form of chronic autoimmune inflammatory condition associated with specific CVD risk factors, development of atherosclerosis is fueled by aberrant response of the innate immune system and overproduction of proinflammatory cytokines [9,10]. A recent progress in characterizing mtROS has led to the generation of a new paradigm, in which blockade of mtROS production may serve as a promising therapy for inhibiting proinflammatory cytokine production and in turn atherosclerosis. Although there were several excellent reviews published 5 years ago in this topic [11,12], new recent discoveries have mandated a more up-to-date and comprehensive review [13-15]. Therefore, in this review we consider current understandings of several compelling questions: 1) how mitochondria generate and dispose of ROS; 2) how production of mtROS is regulated; and 3) what signaling pathways are targeted by mtROS. In addition, we describe the methods to probe mtROS and analyze the merits and flaws of these different methods. Furthermore, we demonstrate how mtROS regulate important vascular function in physiological conditions and activate inflammatory pathways in response to CVD risk factors. In-depth understanding of these processes is critical to developing novel therapeutic drugs against chronic inflammatory conditions such as atherosclerosis.

### Production of mtROS

**Figure 1:**
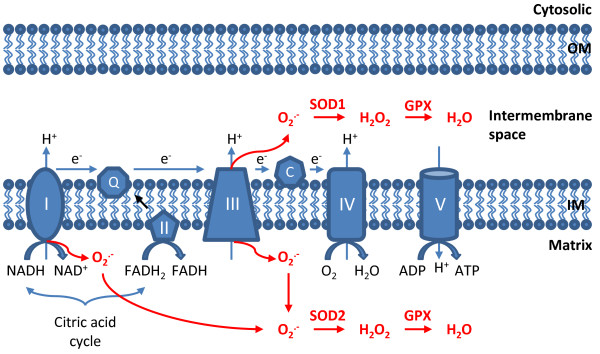
**Production and disposal of mtROS.** Electrons (e^-^) donated from NADH and FADH_2_ pass through the electron transport chain and ultimately reduce O_2_ to form H_2_O at complex IV. MtROS are produced from the leakage of e^-^ to form superoxide (O_2_^  .-^) at complex I and complex III. O_2_^  .-^ is produced within matrix at complex I, whereas at complex III O_2_^  .-^ is released towards both the matrix and the intermembrane space. Once generated, O_2_^  .-^ is dismutated to H_2_O_2_ by superoxide dismutase 1 (SOD1) in the intermembrane space and by SOD2 in the matrix. Afterwards, H_2_O_2_ is fully reduced to water by glutathione peroxidase (GPX). Both O_2_^  .-^ and H_2_O_2_ produced in this process are considered as mtROS. OM: outer membrane; IM: inner membrane.

Mitochondria have a four-layer structure, including outer mitochondrial membrane, intermembrane space, inner mitochondrial membrane and matrix (Figure 1). Generation of mtROS mainly takes place at the ETC located on the inner mitochondrial membrane during the process of oxidative phosphorylation (OXPHOS). Oxidative phosphorylation is an essential cellular process that uses oxygen and simple sugars to create adenosine triphosphate (ATP), which is the cell's main energy source. Five big protein complexes are involved in this process (HUGO Gene Nomenclature Committee Website, Table 1). These ETC complexes are named complex I (NADH dehydrogenase (ubiquinone), 45 protein subunits), complex II (succinate dehydrogenase, 4 protein subunits), complex III (ubiquinol-cytochrome c reductase, 10 protein subunits), complex IV (cytochrome c oxidase, 19 protein subunits), and complex V (ATP synthase, 19 protein subunits). Electrons donated from nicotine adenine dinucleotide (NADH) at complex I and flavin adenine dinucleotide (FADH2) at complex II pass through ETC and ultimately reduce O_2_ to water at complex IV. Meanwhile, positively charged protons (H^+^) are actively being pumped from the mitochondrial matrix into the intermembrane space, resulting in the increased negative charges in the mitochondrial matrix and the upregulated positive charges in the intermembrane space, and thus creating a mitochondrial membrane potential (Δψ_m_) across the inner mitochondrial membrane. This proton-motive force allows complex V - ATP synthase (ATP-ase) to generate ATP from adenosine diphosphate (ADP) and inorganic phosphate when protons re-enter the mitochondrial matrix through the complex V enzyme. However, either by accident or by design, the process of ETC is not perfect. Leakage of electrons at complex I and complex III leads to partial reduction of oxygen to form superoxide (O_2_^  .-^). It has been estimated that 0.2% to 2.0% of O_2_ consumed by mitochondria generates O_2_^  .-^[11]. There are three leak events: complex I leaks O_2_^  .-^ towards the mitochondrial matrix, while complex III leaks O_2_^  .-^ towards both the intermembrane space and mitochondrial matrix [11,16]. Subsequently, O_2_^  .-^ is quickly dismutated to hydrogen peroxide (H_2_O_2_) by two dismutases including superoxide dismutase 2 (SOD2) in mitochondrial matrix and superoxide dismutase 1 (SOD1) in mitochondrial intermembrane space. Collectively, both O_2_^  .-^ and H_2_O_2_ generated in this process are considered as mtROS. These two mtROS have different fates however. Given its electrophilic property and short half-life, O_2_^  .-^ can hardly pass through mitochondrial outer membrane and unlikely to become the candidate of signaling transduction molecule in the cell. Instead, O_2_^  .-^ can undergo radical-radical reaction with nitric oxide (NO) to form peroxynitrite (ONOO_2_^  .-^) within mitochondria, a detrimental oxidant capable of induction of DNA damage, disruption of mitochondrial integrity, and irreversible modification of proteins [11]. In contrast, H_2_O_2_ is electrophobic and more stable than O_2_^  .-^. Indeed, the concentrations of H_2_O_2_ in mitochondria are 100 times greater than that of O_2_^  .-^[17]. These properties render mitochondrial H_2_O_2_ an ideal signaling molecule in the cells.

**Table 1:** Mitochondrial DNA (mtDNA) and nuclear DNA (nDNA) encoded subunits for the electron transport chain

**Complex**						
Genome	I	II	III	IV	V	Total
mtDNA	7	0	1	3	2	13
nDNA	38	4	9	16	17	84
Total	45	4	10	19	19	97

Source: http://www.genenames.org/genefamilies/mitocomplex#coml.

### Scavenging of mtROS

Owing to the high reactivity and toxicity of mtROS, mammalian cells have evolved a number of antioxidant enzyme systems to scavenge mtROS as soon as they are generated. As mentioned in the previous section, the SOD family of antioxidant enzymes catalyze the dismutation of O_2_^  .-^ to H_2_O_2_. Subsequently, H_2_O_2_ is quickly reduced to water by two other enzymes, catalase and glutathione peroxidase (GPx) (Figure 1). It should be noted that all the mitochondrial antioxidant enzymes are encoded by the nuclear genome but not mitochondrial genome, and these enzymes are subsequently imported into the mitochondria after their protein translation.

### SODs

Three isoforms of SOD have been identified, including SOD1/copper-zinc SOD (CuZn-SOD), SOD2/manganese SOD (Mn-SOD), and extracellular SOD3 (EC-SOD). SOD1 is widely distributed throughout the cell cytoplasm, nucleus, and intermembrane space of mitochondria [18]. SOD2 is expressed only in the mitochondrial matrix [18], and SOD3 is found in the extracellular space. The physiological importance of SOD2 is high-lighted by the finding that in contrast to other SOD isoforms, the deficiency of SOD2 causes early neonatal death in gene knockout mice [19] and endothelial dysfunction in carotid artery of proatherogenic apolipoprotein E (ApoE)-deficient mice [11,20].

### Catalase

Catalase is a heme-containing tetramer of four polypeptide chains that reduces H_2_O_2_ to water. Although catalase is highly efficient at reducing hydrogen peroxide, it may not play a central role in scavenging ROS in the mitochondria since it is localized mainly in peroxisomes except that rat heart mitochondria does partially depend on catalase to scavenge ROS [21]. Nevertheless, overexpression of catalase in ApoE−/− mice results in the retardation of atherosclerosis [22]. In addition, overexpression of catalase in the mitochondria decreased oxidative damage, inhibited cardiac pathology, and extended the lifespan of mice [23]. These results suggest the importance of catalase in suppressing cardiovascular inflammation and damage and atherosclerosis.

### GPx

GPx catalyzes the reductive inactivation of H_2_O_2_ using reduced glutathione (GSH) as a cofactor. GSH is a tripeptide containing of three amino acid residues including glutamate, cysteine, and glycine. During the process of reducing H_2_O_2_, GSH is oxidized to oxidized glutathione (GSSG). GSSG is then recycled back to GSH by the enzyme glutathione reductase (GR) using NADPH as a substrate [13]. Thus, the maintenance of GSH for optimal scavenging capacity is dependent on the bioavailability of NADPH stores. Deficiency of GPx results in acceleration of atherogenesis in ApoE−/− mice, highlighting the importance of glutathione peroxidase in suppressing vascular inflammation and atherosclerosis [24].

### Peroxiredoxin

Peroxiredoxins are a family of antioxidant enzymes that regulate cytokine-induced peroxide levels and mediate signal pathways [25]. There are six peroxiredoxins in this family. Importantly, H_2_O_2_ has the highest affinity to peroxiredoxin 2 (100%), then to GSH (<0.01%), to Cdc25B (<0.0001%) and to protein tyrosine phosphatase 1B (<0.000001%), demonstrating the importance of peroxiredoxins [1]. Overexpression of mitochondrial matrix peroxiredoxin (peroxiredoxin-3) prevents left ventricular remodeling and failure after myocardial infarction in mice [26].

### Thioredoxins

Thioredoxins are small proteins that play a variety of roles depending upon binding interactions and oxidoreductase activity. Mammalian thioredoxin-2 (Trx2) is a mitochondrial protein. Trx2 deficiency results in embryonic lethal at gestational day 10.5 and embryos show massive apoptosis. The timing coincides with the maturation of mitochondrial function. In addition, Trx2 protects against vascular pathology in the ApoE-knockout mouse model for CVD [27]. Furthermore, the accumulated data strongly support a role for Trx2 in protecting against oxidant-induced apoptosis via regulating mitochondrial permeability transition [28].

### Synthetic mtROS scavengers

Although several natural antioxidants including vitamin E have been shown to decrease mtROS; however their effectiveness was limited as they did not accumulated within mitochondria nor could they cross the blood–brain barrier efficiently [29,30]. To address this issue, several synthetic mtROS scavengers have been developed. These compounds easily pass through all biological membranes, including the blood–brain barrier, into cells and tissues affected by mtROS [31]. The first targeted ROS scavenger is MitoVit-E in which vitamin E is covalently attached to a triphenylphosphonium cation [32]. MitoVit-E decreases ROS production and apoptosis in aortic endothelial cells induced by oxidative stress, but it is ineffective against hypoxic–ischemic striatal injury in neonatal rats [33]. The second ROS scavenger is MitoQ10 which consists of a lipophilic triphenylphosphonium cation covalently attached via an aliphatic linker to a ubiquinone derivative [34]. After detoxifying an oxidant species, MitoQ10 can be regenerated by the respiratory chain. MitoQ10 inhibits mitochondrial oxidative damage in rodent models of cardiac ischemia and reperfusion injury [33]. Moreover, the results of animal studies using MitoQ10 or an alternative compound termed SkQ1 are promising. When perfused through isolated heart preparations or fed to rats, SkQ1 is able to reduced ischemia-induced arrhythmia and infarct size; SkQ1 is used at a concentration that is an astounding one million fold lower than that of MitoQ. The results of ongoing clinical studies are awaited with great interest [33].

### Regulation of mtROS

**Figure 2:**
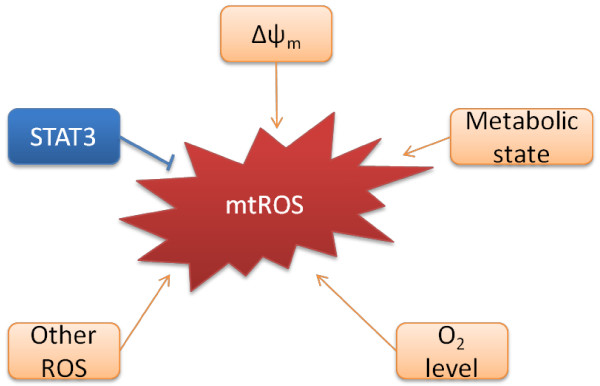
**Regulation of mtROS production.** A number of factors including mitochondrial membrane potential (Δψ_m_), metabolic state of mitochondrial, O_2_ concentration regulate the production of mtROS. Non-mitochondrial generated ROS can also augment mtROS production, a process known as “ROS-induced ROS”. Meanwhile, transcription factor STAT3 has recently been found to suppress mtROS production independent of its nuclear factor activity.

ROS production in mitochondria is determined by the rates of both mtROS production and disposal, and it is regulated by a number of factors, such as mitochondrial membrane potential, metabolic state of mitochondria, and O_2_ levels [35] (Figure 2).

### Mitochondrial membrane potential (Δψ_m_)

As described above, Δψ_m_ is created when protons are pumped from the mitochondrial matrix to the intermembrane space as electrons pass through the ETC. The concept that higher (more polarized) Δψ_m_ is associated with greater mtROS generation is widespread in literature, and this is thought to be due to the slowed electron transport [11,20]. This idea is supported by the observation of decreased ROS generation when Δψ_m_ is dissipated by either chemical uncouplers [36], such as carbonyl cyanide p-(tri-fluromethoxy)phenyl-hydrazone (FCCP) [37], or overexpression of mitochondrial uncoupling proteins (UCPs) [38]. However, it has also been shown that uncoupling of the mitochondrial ETC in cardiomyocytes using chemical uncouplers in fact increased ROS accumulation [15]. To reconcile this obvious discrepancy, a redox-optimized ROS balance hypothesis is proposed, stating that physiological ROS signaling occurs within an optimized mitochondrial membrane potential, and oxidative stress can happen at either the extreme of high Δψ_m_ or low Δψ_m_[15]. This hypothesis is based on the fact that the redox couples involved in substrate oxidation (NADH) are closely linked to the redox couples involved in antioxidant defenses (NADPH). Hence, it is vital to balance an adequate level of Δψ_m_ to maintain matrix NADPH rather than NADP^+^, which is necessary for mitochondrial antioxidant enzyme systems. In other word, an increase in mitochondrial uncoupling of the ETC can increase ROS production primarily because the antioxidant system of the cell is compromised.

### Metabolic state of mitochondria

Metabolic state of mitochondria is another important factor that modulates mtROS production. The concept of metabolic states of mitochondria was proposed by Britton Chance and G.R. Williams in 1955 [39]. State I is the first respiratory state observed when isolated mitochondria are added to mitochondrial respiration medium containing oxygen and inorganic phosphate, but no ADP and no reduced respiratory substrates [39]. In State I, leak respiration may be supported to some extent by undefined endogenous substrates, which are oxidized and slowly exhausted. State II is the substrate-limited state of residual oxygen consumption, after addition of ADP to isolated mitochondria suspended in mitochondrial respiration medium in the absence of reduced substrates [39]. State III respiration is the ADP-stimulated respiration of isolated coupled mitochondria in the presence of high ADP and phosphate concentrations, supported by a defined substrate or substrate combination at saturating oxygen levels [39]. State IIIu (u for uncoupled) has been used frequently in bioenergetics on the fundamental difference between OXPHOS capacity and non-coupled ETS capacity. State IV is the respiratory state obtained in isolated mitochondria after State III, when added ADP is phosphorylated completely to ATP driven by electron transfer from defined respiratory substrates to O_2_[39]. State V is the respiratory state obtained in a protocol with isolated mitochondria after a sequence of State I to State IV, when the concentration of O_2_ is depleted in the closed oxygraph chamber and zero oxygen (the anaerobic state) is reached [39]. The State V is defined in the original publication in two ways - State V may be obtained by antimycin A treatment and by anaerobiosis. Resting mitochondria (State IV) are characterized by low electron flow and ATP synthesis, low rates of O_2_ consumption, and high NADH/NAD^+^ ratio leading to high ROS production. When mitochondria are synthesizing ATP (State III), the opposite happens (high electron flow and ATP synthesis, high rates of O_2_ consumption, high NADH/NAD^+^) which results in lower ROS production [40].

Importantly, endogenous modulators such as NO and Ca^2+^ can regulate the production of mtROS by regulating the metabolic states of mitochondria. NO is a diffusible gas synthesized by three NOS enzymes including endothelial NOS (eNOS), inducible NOS (iNOS) and neuronal NOS (nNOS). These three enzymes share 50–60% homology at the amino acid sequence and have an N-terminal oxygenase domain with heme-, L-arginine-, tetrahydrobiopterin (BH4)-binding domains, a central calmodulin (CaM)-binding region, and a C-terminal reductase domain with NADPH, FAD, and FMN binding sites [41]. The identification of NOS in the mitochondria [42] and the fact that the ETC has several NO^.^ reactive-redox metal centers [11] strongly argue NO’s role as an important modulator of mtROS production. NO can modulate mitochondrial respiration and oxygen consumption through reversible binding and inhibition at complex IV, leading to the accumulation of NADH and increases in ROS production [43]. Mitochondria also participate in Ca^2+^ homeostasis by serving as a high-capacity, low-affinity transient Ca^2+^ store. Unlike NO, it seems that a moderate increase in mitochondrial Ca^2+^ stimulates the rate of electron flow in the ETC and thus decreases mtROS generation [44]. However, it should be noted that mitochondrial Ca^2+^ overload increases mtROS production, which is independent of the metabolic states of mitochondria [45].

### O_2_ concentration

MtROS production also depends on O_2_ concentration. As cellular O_2_ concentration increases, the rate of mtROS production increases linearly [46]. However, during hypoxia, a paradoxical increase in mtROS release was reported [47]. This mtROS release appears to come from complex III and functions as a regulator of hypoxia-inducible factor 1α (HIF-1α). Nevertheless, the precise molecular basis underlying the seemingly controversial relationship between ambient oxygen levels and mtROS production remains obscure. The redox-optimized ROS balance hypothesis mentioned previously can be used to account for this discrepancy. It is postulated that hypoxic cells would exhibit high Δψ_m_ and augmented mtROS production due to the low electron flow [15]. In this setting, the increased generation of mtROS could then be relieved by overexpressing mitochondrial UCPs [48].

### Mitochondrial mass

MtROS may be additionally altered by mitochondrial mass. Theoretically, the level of mtROS generation should be positively correlated to the quantity of mitochondria in the cell. Nevertheless, it has been shown that the mitochondrial biogenesis factor peroxisome-proliferator-activated receptor-γ coactivator 1α (PGC1α) not only increases mitochondrial mass but also increases the expression of many antioxidant enzymes including GPx and SOD2 [49]. It therefore stands to reason that mitochondrial mass is not an important factor that regulates mtROS production.

### Mitochondrial fusion

Mitochondria are dynamic organelles which frequently change their number, size, shape, and distribution in response to intra- and extracellular stimuli. After proliferated from pre-existing ones, fresh mitochondria enter constant cycles of fission and fusion that can be classified into two distinct states - individual state and network state. When compromised with various injuries, solitary mitochondria are subjected to organelle degradation, which relies on autophagy, a self-eating process that plays key roles in manifold cell activities. Recent reports reveal that defects in autophagic degradation selective for mitochondria (mitophagy) are associated with neurodegenerative diseases, highlighting the physiological relevance of mitophagy to cellular functions [50]. The fission and fusion processes are important for mitochondria to redistribute their proteins, protecting the cells from the harmful effects of mitochondrial DNA (mtDNA) mutations. These processes are regulated by *N*-ethylmaleimide-sensitive factor attachment protein receptor (SNARE)-like proteins which include mitofusin-1 and −2 [51]. Whether mtROS regulate mitochondrial fusion remains unclear, however it has been reported that mtROS enhance mitochondrial fragmentation [52].

### Transcription factors

Several nuclear transcription factors (TFs) with well-characterized functions in the nucleus are also present in the mitochondria and become mitochondrial TFs (mitoTFs). MitoTFs include those of the nuclear hormone receptor family as well as TFs such as p53, nuclear factor kappa B (NF-κB) and the signal transducer and activator of transcription (STATs) that are activated downstream of the binding of growth hormones and cytokines to cell-surface receptors [53]. These TFs have several different mechanisms in regulating mitochondrial function and ROS levels. P53 can bind to the Bcl-2 family members and induces apoptosis. Also, p53 can inhibit SOD2. In addition, interferon regulatory transcription factor (IRF) family 3 (IRF3) can interact with proapoptotic protein Bax. Moreover, TFs including cAMP-responsive transcription factor (CREB), NF-κB, myocyte enhancer factor-2D (MEF2D) and STAT3 all can regulate gene expression.

STAT3, initially identified as a transcription factor that regulates gene expression in response to cytokines such as interleukin (IL)-6 and IL-10, has recently been found to modulate mtROS through mechanisms independent of its nuclear factor activity, but dependent on its ability to directly modulate the activity of the ETC [13,54]. It has been shown that STAT3 is present in the mitochondrial matrix, and deficiency of STAT3 in murine hearts leads to decreased activities of complexes I and II while increasing mtROS at complex I [13]. However, the molecular mechanism by which STAT3 modulates ETC activity is not well understood and it remains to be determined whether STAT3 is unique among STAT proteins in localizing to mitochondrial matrix and regulating mtROS. Since STAT3 responds to cytokines of the IL-6 and IL-10 families, which themselves regulate cellular metabolism process, it is tempting to speculate that by modulating mitochondrial ETC activity and mtROS generation, STAT3 links cytokine signaling pathways to cellular metabolism.

HIF-1 mediates adaptive responses to chronic hypoxia with reduced oxygen availability by regulating gene expression. HIF-1 reduces mtROS production under hypoxic conditions by multiple mechanisms including: *i*) a subunit switch in cytochrome c oxidase from the cytochrome c oxidase subunit IV (COX4)-1 to COX4-2 regulatory subunit that increases the efficiency of mitochondrial complex IV; *ii*) induction of pyruvate dehydrogenase kinase 1, which shunts pyruvate away from the mitochondria; *iii*) induction of BCL2/adenovirus E1B 19 kDa protein-interacting protein 3, which triggers mitochondrial selective autophagy; and *iv*) induction of microRNA-210, which blocks assembly of Fe/S clusters that are required for oxidative phosphorylation [55].

### Epigenetic regulatory enzyme protein deacetylases

Recent progress has demonstrated that the Class III (NAD + −dependent) deacetylases termed sirtuins play a critical role in suppressing inflammation. Specifically, the subcellular locations of these sirtuins including the nucleus (SIRT-1,-2,-6,-7), cytosol (SIRT-1,-2) and mitochondria (SIRT-3,-4,-5) [56] suggest the important functions of these Sirtuins in these locations [33,57]. Endothelial overexpression of SIRT1 has been shown to suppress atherosclerosis and maintain normal endothelial function in mice fed a high-fat diet [58] and can also protect against hyperglycemia-induced vascular cell senescence [59]. Recent progress has demonstrated that sirtuins’ complicated regulatory functions can be activated by resveratrol, an antioxidant polyphenol compound isolated from grape skin. Resveratrol has shown promising clinical benefits as anti-aging [60], anti-inflammatory [61], anti-diabetic [62], anti-viral [63], anti-neoplastic [64], and anti-CVD agents [65]. Several mechanisms underlying sirtuins functions have been found, for example, Sirt1 induces eNOS function and promotes NO generation. Sirt1 inhibits type 1 angiotensin receptor and enhances tissue inhibitor of metalloproteinase. Sirt1 also induce superoxide dismutase and other antioxidant genes and suppress cellular burden of ROS.

### Cytokines

As early as a decade ago, mtROS were already found to be induced by tumor necrosis factor (TNF)-α which is mediated by ceramide-dependent signaling pathways [66]. However, mtROS do not appear to have a role in TNF-α triggered NF-κB activation and ICAM-1 expression in endothelial cells [67]. It is later unveiled that TNF-α induces a calcium-dependent increase in mtROS that causes the shedding of TNF-α receptor-1 and reduces the severity of microvascular inflammation [68]. Another proinflammatory cytokine, interferon-γ (IFN-γ) is capable of upregulating the expression of many nuclear genes encoding mitochondrial ETC and inducing mtROS by activation of estrogen-related receptor alpha (ERRα) and coactivator peroxisome proliferator-activated receptor gamma coactivator-1 beta (PGC1β).

Adipose tissue is not only an organ of energy storage but also an endocrine organ capable of producing a number of cytokines termed as adipokines [69]. Two members of the adipokine family, leptin and resistin, have been shown to increase mtROS production [70,71]. Leptin is a circulating adipokine involved in the control of body weight. Not surprisingly, leptin could “talk” with mitochondria and induces mtROS by increasing fatty acid oxidation via protein kinase A (PKA) activation [70]. Resistin is another adipocyte-derived cytokine that play an important role in insulin resistance, adipogenesis, and inflammation. By reducing mitochondrial Δψ_m_ and activities of antioxidants including catalase and SOD, resistin could induce eNOS downregulation through overproduction of mtROS in endothelial cells [71].

A recent report showed that CD8+ memory T cells, but not CD8+ T effector (Teff) cells, possess substantial mitochondrial spare respiratory capacity (SRC). SRC is an extra capacity available in cells to produce energy in response to increased stress or work, which is associated with cellular survival. Interleukin-15 (IL-15), a cytokine critical for CD8+ memory T cells, regulates SRC and oxidative metabolism by upregulating mitochondrial biogenesis and expression of carnitine palmitoyl transferase (CPT1a), a metabolic enzyme that controls the rate-limiting step to mitochondrial fatty acid oxidation (FAO). These results demonstrate how cytokines control the bioenergetic stability of memory T cells after infection by regulating mitochondrial metabolism [72]. In addition, IL-15 transgenic mice run twice as long as control littermates in a run-to-exhaustion trial and preferentially uses fat for energy metabolism. Skeletal muscles in IL-15 transgenic mice have high expression of intracellular mediators of oxidative metabolism that are induced by exercise, including sirtuin 1, peroxisome proliferator-activated receptor (PPAR)-δ, PPAR-γ coactivator-1α, and PPAR-γ coactivator-1β [73].

### Non-mitochondrial ROS sources

Under certain conditions, non-mitochondrial generated ROS can augment mtROS production, a process known as “ROS-induced ROS”. It has been demonstrated that many other ROS-producing enzymes, including NADPH oxidase [74], xanthine oxidase [75], and uncoupled eNOS [76], can stimulate mtROS production. The “ROS-induced ROS” system downstream of angiotensin II (Ang II) signaling pathway is well characterized. Ang II is a well-known stimulator of NADPH-oxidase-derived ROS [77], but a role of mtROS downstream of Ang II-mediated cellular signaling has also emerged recently [74]. In fact, it has been suggested that by activating NADPH oxidase, Ang II induces mtROS, which in turn leads to further activation of NADPH oxidase. Moreover, scavenging of mtROS using mitochondria-targeted antioxidant can interrupt this vicious cycle and significantly decrease blood pressure after the onset of Ang II-induced hypertension [78].

The question of how other sources of ROS induce mtROS remains. However, the importance of p66^shc^ in this process is highlighted by the fact that p66^shc^ is localized within the mitochondrial intermembrane space and can directly transfer electrons from *cytochrome c* to O_2_ to generate mtROS [79]. Importantly, intracellular antioxidants such as GSH are thought to maintain the mitochondrial form of p66^shc^ in an inactive state [80]. Thus, p66^shc^ may serve as a thiol-based redox sensor that signals to mitochondria to induce mtROS when the ROS level in cytoplasm becomes high. As such, the atherosclerotic risk factor, oxidized LDL, activates p66^shc^ through NADPH oxidase [81]. Furthermore, deficiency of p66^shc^ gene renders mice resistant to complications of atherosclerosis [82].

### MtROS levels and signaling of mtROS

**Figure 3:**
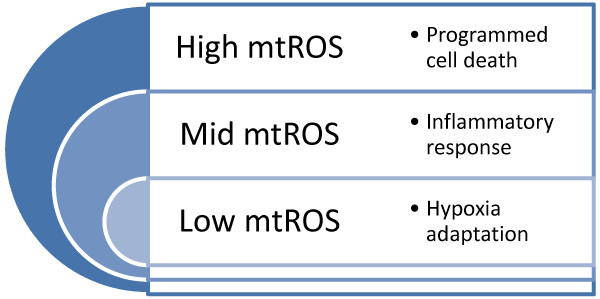
**Signaling of mtROS.** At low levels, mtROS participate in the process of hypoxia adaptation by regulating the stability of hypoxia-inducible factor 1α (HIF-1α); moderate levels of mtROS are involved in regulating the production of proinflammatory cytokines by directly activating the inflammasome and mitogen-activated protein kinase (MAPK); high levels of mtROS are capable of inducing apoptosis and autophagy by oxidation of the mitochondrial pores and autophagy-specific gene 4 (ATG4) respectively.

**Figure 4:**
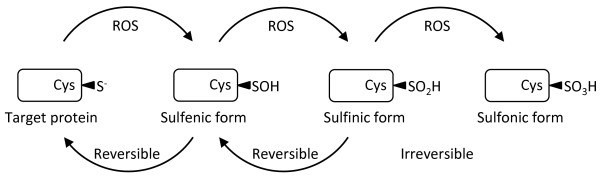
**Modification of proteins by ROS.** ROS can oxidize specific reactive cysteine (Cys) residues within target proteins generating sulfenic form (RSOH) of proteins. RSOH is unstable and can be further oxidized to sulfinic form (RSO_2_H). Under greater oxidative stress, sulfonic form (RSO_3_H) can be generated. Although the formation of RSOH and RSO_2_H is reversible, generation of RSO_3_H is irreversible.

Once thought as merely the by-products of cellular metabolism, mtROS are increasingly viewed as important signaling molecules [83]. At low levels, mtROS are considered to be important for metabolic adaptation as seen in hypoxia. Moderate levels of mtROS, stimulated by danger signals such as Toll-like receptor 4 ligand bacterial endotoxin lipopolysaccharide (LPS), are involved in regulating inflammatory response. Finally, high levels of mtROS activate apoptosis/autophagy pathways capable of inducing cell death [83] (Figure 3). But, how does mtROS signal in the cell? Much like the events of phosphorylation modification of proteins, mtROS promote cell signaling via the oxidation of certain reactive cysteine residues of proteins [84]. Cysteine residues can exist in a number of oxidative states, including sulfenic form (RSOH), sulfinic form (RSO_2_H), and sulphonic (RSO_3_H) form. Although the pKa of most thiol group on free cysteine is between 8 and 9, the surrounding environment of certain reactive cysteine residues can be substantially modified to result in reduced pKa as low as 4 to 5. These reactive cysteine residues (RS^-^) are easily oxidized to RSOH. RSOH is unstable and can undergo further oxidation into RSO_2_H. Furthermore, under greater oxidative stress than that for generating RSOH and RSO_2_H, RSO_3_H is generated. Although the generation of RSOH and RSO_2_H is readily reversible, formation of RSO_3_H is irreversible (Figure 4). Using computational methods and proteomic approaches, it is suggested that RS^-^ might exist in more than 500 proteins, allowing mtROS to modulate a wide variety of protein targets in the cells [85,86].

### Low mtROS

Accumulating evidence suggests that mtROS released under hypoxic conditions regulates HIF-1α. HIF-1α is a heterodimeric protein composed of an α subunit and a β subunit [87], the latter being constitutively expressed. The stability of the α subunit, however, is regulated by oxygen levels such that, it is stabilized under hypoxic conditions while it undergoes proteasomal degradation under normoxic conditions [83]. The picture is becoming clear that, HIF-1α is stabilized in response to mtROS and then feedback and inhibit the production of mtROS [83]. The latter feedback activity is suggested by recent identification of a mitochondrial gene, NADH dehydrogenase [ubiquinone] 1 alpha subcomplex, 4-like 2 (NDUFA4L2) that serves as a direct HIF-1α target [88]. Employing NDUFA4L2-silenced and NDUFA4L2 knockout cells, it has been demonstrated that inhibiting mtROS generation via NDUFA4L2 upregulation induced by HIF-1α is an essential cellular adaption process during hypoxia.

### Moderate mtROS

Several recent studies unveil the fact that mtROS act as essential signaling molecules that regulate inflammatory process. On one hand, one member of the cytosolic nucleotide binding and oligomerization domain (NOD)-like receptor (NLR) family, pyrin domain containing 3 (NLRP3)-containing inflammasome (caspase-1 activating protein complex) is shown to be activated by mtROS [5]. The NLRP3 inflammasome is a multiprotein complex consisting of the sensor protein NLRP3, the adaptor protein ASC, and the inflammatory protease precursor pre-caspase-1 [89]. Although several sensor proteins including NLRP3, NLRC4 (NLR family, CARD-containing 4), AIM2 (Absent in melanoma 2), and NLRP6 (NOD-like receptor family pyrin domain containing 6) have been shown to form inflammasomes with caspase-1, the NLRP3 inflammasome has drawn the most attention due to its association with the onset and pathogenesis of numerous inflammatory diseases [90]. Conformational changes in NLRP3 lead to the assembly of the inflammasome and activation of caspase-1, which promotes the maturation and secretion of proinflammatory cytokines IL-1β and IL-18. Besides its role in regulating inflammatory processes, the NLRP3 inflammasome also drives a form of inflammatory cell death, termed pyroptosis [91]. In contrast to non-inflammatory apoptosis, pyroptosis causes local inflammation, as it is linked to the caspase-1 cleavage of pre-IL-1 β and pre-IL-18 and release of mature IL-1β and IL-18 [92]. While apoptotic cells retain their plasma membrane integrity until the final stage of apoptosis, pores rapidly form in the plasma membrane of pyroptotic cells. Such pores provide a direct route for the release of inflammatory molecules including IL-1β and IL-18 [93]. Of note, it has been shown that autophagy blockage results in accumulation of dysfunctional, mtROS generating mitochondria, which also activates the NLRP3 inflammasome [5]. In addition, both NLRP3 and its adaptor ASC co-localize with endoplasmic reticulum (ER) and mitochondria upon inflammasome stimulation. However, it remains unknown whether NLRP3 is the direct target of mtROS. Indeed, this idea is challenged by the observation that ROS inhibitors block priming instead of activation of the NLRP3 inflammasome [94]. Priming of the NLRP3 inflammasome controls the threshold of the inflammasome activation, which involves induction of pro-IL-1β and NLRP3 expression [89]. In this regard, mtROS may be involved in induction of NLRP3 transcripts upstream of post-translational NLRP3 activation. Based on the differences of tissues in the expression levels of NLRP3 and other inflammasome components, we proposed novel “three-tier model and inflammation privilege” for explaining the readiness of tissues in the onset of inflammation in response to stimuli [95].

On the other hand, it has been shown that mtROS cause oxidation and inactivation of MAPK phosphatases, which results in sustained MAPK activation [96]. In addition, inhibition of mtROS production attenuates MAPK activation and production of IL-6 induced by LPS whereas macrophages lacking inflammasome components produce normal levels of IL-6 after LPS stimulation comparable to that of wild-type macrophages [97]. Thus, in a critical mechanistic divergence, mtROS could also regulate inflammasome-independent proinflammatory cytokines such as IL-6 by affecting transcription factor pathways. Indeed, mtROS also appear to activate NF-κB and induce the upregulation of cell surface adhesion molecules in endothelial cells as a part of endothelial cell activation program [67,98].

### High mtROS

Similar to the caspase-1 activating inflammasomes, activation of the structurally related protein complex termed apoptosome also requires mtROS [99]. Apoptosome is an oligomeric structure that is assembled when apoptosis activating factor (APAF)-1 interacts with *cytochrome c* released from mitochondria, and the activation of apoptosome initiates apoptosis by recruiting and activating pre-caspase-9 [100]. MtROS contribute to apoptosome activation by oxidation of the mitochondrial pores, which leads to *cytochrome c* release [99]. If mtROS are involved in both inflammasome and apoptosome activation, what mechanisms regulate the choice of the signaling pathways? One possibility is that the intensity or duration of mtROS release determines the ultimate biological outcome, with high levels of mtROS capable of inducing apoptotic cell death.

Recent evidence yet suggests the involvement of mtROS in the induction of autophagy, another form of programmed cell death [101]. Starvation of cells stimulates formation of ROS, which localizes with mitochondria. Treatment of cells with antioxidant agents abolishes starvation-induced autophagy. Moreover, a specific reactive cysteine residue on autophagy regulatory protein, autophagy-specific gene 4 (ATG4) is shown to be ROS-sensitive. This new working model in which mtROS oxidize ATG4 and induce autophagy requires further examination.

### Detection of mtROS by fluorescent probes

Recent progress on characterization of mtROS has been greatly benefited from new advances in using fluorescence probes in the detection of mtROS. Fluorescent dyes including dichlorodihydrofluorescein (DCF) and dihydroethidium (DHE) have been widely used to detect intracellular ROS in early studies. While DCF is the most widely used probe for detecting intracellular H_2_O_2_, DHE is the most frequently used fluorescent indicator for O_2_^  .-^. In conjugation with mitochondria-specific markers such as MitoTracker using confocal microscopy, one can determine whether ROS are generated from mitochondria [102-104]. To minimize the effect in subtracting the fluorescence signals coming from non-mitochondrial organelles, some fluorescence indicators have been modified to target mitochondria specifically. The most common way is through the use of lipophilic cations, which are attracted to the negative potential environment caused by the proton gradient across the inner mitochondrial membrane.

MitoSOX is a triphenylphosphonium (TPP+)-linked DHE compound. It exploits the steep electrochemical gradient across the mitochondrial inner membrane to enrich the TPP-tag fluorescence more than a 100-fold within the mitochondria compared with the cytosol [105]. MitoSOX has been effectively used to directly detect mitochondria-derived O_2_^.-^ in various cell types [106]. However, the reactions of both DHE and MitoSOX with ROS yield two fluorescent products, one of which is O_2_^.-^ specific, while the other is formed in response to general oxidative stress. Thus, fluorescence microscopy or related flow cytometric techniques are not sufficient to measure the superoxide-specific hydroxylated products using both classical DHE and novel MitoSOX. High-performance liquid chromatography (HPLC) methodologies are required to separate and identify these products [78,107] (Table 2).

**Table 2:** Comparison of different detection methods of mtROS

**Methods**	**Drugs**	**Detected species**	**Principle**	**Limits**
Fluorescent probes	MitoSOX	Superoxide	Attaching a hydrophobic cation to dihydroethidium.	Two fluorescent products, one of which is superoxide specific. The two products need to be separated by HPLC.
	MitoPY1	Hydrogen peroxide	Attaching a hydrophobic cation to a phenylboronate ester.	It also reacts with peroxynitrite.
	MitoAR MitoHR	Hydroxyl radical	Reaction with hydroxyl radical cleaves off the quenching motif.	They also react with peroxynitrite.
Mitochondrial inhibitors	Rotenone Antimycin	General ROS	Rotenone is complex I inhibitor and antimycin is complex III inhibitor.	Rotenone either decrease or increase ROS; Interruption of the ETC.
Mitochondrial antioxidant	MitoQ MitoTEMPO MitoVitE	General ROS	Attaching a hydrophobic cation to antioxidants.	-
Electron spin resonance	MitoDEPMPO	Free radicals	Free radicals have unpaired electrons, and thus are paramagnetic and detectable by ESR.	Lower sensitivity than fluorescent probes.

Appending a TPP + motif to a phenylboronate ester generates MitoPY1 [108], which is a biologically compatible probe for detecting mitochondria-derived H_2_O_2_. MitoPY1 selectively responds to rises in H_2_O_2_ levels by a significant fluorescence increase detected by both confocal microscopy and flow cytometry methods [108]. It should be noted though: the phenylboronate moiety in MitoPY1 also reacts with ONOO^-^, so MitoPY1 will respond to this molecule as well.

Highly reactive oxygen species (hROS), including hydroxyl radical (^.^OH), hypochlorous acid (HOCl), and ONOO^-^, are generated secondary to mtROS formation (O_2_^  .-^, H_2_O_2_) [109]. They are highly toxic by directly oxidizing nucleic acids, proteins, and lipids in the cell. ^.^OH originates from H_2_O_2_ through Fenton chemistry in the presence of iron or copper centers which are prevalent in the mitochondria. Likewise, NO produced by mitochondrial NOX can combine with O_2_ ^-^ to form ONOO^-^. In addition, myeloperoxidases (MPOs) catalyze the reaction of H_2_O_2_ into HOCl, which possibly could diffuse into and damage mitochondria. As such, several probes have been developed to detect these mitochondrial hROS. For example, MitoAR and MitoHR are developed by attaching rhodamine-like fluorophores (which functions similarly as TPP motif) to either a 4-amino-phenyl aryl ether or a 4-hydroxy aryl ether group, respectively. The ether motif on both MitoAR and MitoHR quench the fluorescence emission. Reaction with mitochondrial hROS cleaves off the quenching motif and results in the highly fluorescent rhodamine type reporters [110]. MitoAR mainly detects ^.^OH and HOCl while MitoHR is most sensitive to ^.^OH, while both of these two probes also react with ONOO^-^ in a slower rate.

### Detection of mtROS by other methods

#### ETC inhibitors and mitochondria-targeted antioxidant

Combination of traditional ROS detection method (DHE fluorescence) with ETC inhibitors can also help to identify mtROS [111-114]. This approach is instrumental, but the results obtained from these inhibitors are not always consistent. For example, when intact cells are used, complex I inhibitor rotenone has been shown to either decrease or increase ROS production [104,115]. Another potential problem with mitochondrial inhibitors is their interruption of the ETC, which may alter cell metabolism such as ATP synthesis. In this regard, mitochondrial-targeted antioxidants represent a promising tool in studying mtROS. A series of antioxidants have been chemically bonded to TPP to scavenge mtROS, including α-tocopherol (MitoVitE) [104], nitroxides (MitoTEMPO) [78], and ubiquinol (MitoQ) [114]. Among these, MitoQ is the best characterized mitochondria-targeted antioxidant in animal studies [34,105,116]. The ubiquinol form of MitoQ is oxidized by mtROS to the ubiquinone form, which is quickly re-reduced by complex II in the ETC, restoring its mtROS scavenging capacity. In addition, the oral administration of MitoQ is safe for at least 24 weeks in mice and rats [117]. For these reasons, orally administered MitoQ has been tested in a number of in vivo animal studies and shown protection against oxidative stress in a variety of CVD, including: hypertension [118], cardiac ischemia reperfusion (I/R) injury [119], kidney damage in type I diabetes [120], sepsis [121,122], and endothelial damage by nitroglycerin [123].

### ESR

In some experiments, electron spin resonance (ESR) spectroscopy is used to study mtROS [74,124]. Free radicals such as superoxide have unpaired electrons, and thus are paramagnetic and detectable by ESR. The application of different spin probes, cell permeable versus nonpermeable, allows one to localize and distinguish mtROS versus non-mtROS and intracellular versus extracellular ROS. Another advantage of ESR assay is that cells or tissue samples can be frozen and analyzed later. In addition, ESR has potential in determining the chemical identities of ROS. For example, MitoDEPMPO has been developed as a mitochondria-targeted nitrone traps for ESR detection of mitochondria-derived superoxide [125]. Moreover, it can be used to analyze cells in suspension. However, the sensitivity of ESR assay is lower than that of methods based on fluorescence. Moreover, ESR assay cannot be applied for ROS detection and determination of intracellular distribution in a single cell (Table 2).

### MtROS in vascular cells

#### Endothelial cells (ECs)

Even when plenty of oxygen is presented, ECs rely heavily on glycolysis rather than OXPHOS-mitochondrial respiration to generate ATP [126]. Nevertheless, ECs still contain functional mitochondria, in which oxidative phosphorylation continues [127,128]. Thus, the primary function of mitochondria in ECs may be the regulated generation of ROS for cells signaling purpose, but not generation of ATP for energy production. If this is true, ECs could modulate mtROS production without jeopardizing their energy needs. Indeed, mtROS are involved in regulating a variety of important endothelial functions under basal conditions whereas activating proinflammatory pathways in response to cardiovascular risk factors in ECs [129].

**Figure 5:**
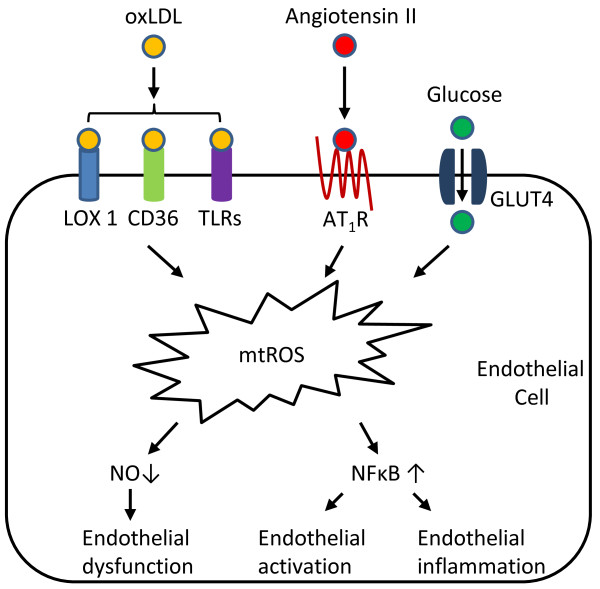
**Role of endothelial mtROS in atherosclerosis.** Pathologic stressors as diverse as oxidized low-density lipoproteins (oxLDL), glucose, and Angiotensin II are all capable of inducing mtROS in endothelial cells through their receptors. Excessive mtROS then directly bind to NO and induce endothelial cell dysfunction. Overproduction of mtROS also leads to activation of proinflammatory transcription factors such as nuclear factor kappa B (NFκB). This in turn increases the expression of adhesion molecules and production of inflammatory cytokines in endothelial cells, both of which contribute to the development of atherosclerosis. LOX-1: lectin-type oxidized LDL receptor 1; TLRs: Toll-like receptors; AT_1_R: Angiotensin II receptor, type 1; GLUT4: glucose transporter type 4.

Under normal physiological conditions, mtROS are capable of regulating vascular homeostasis. Firstly, it has been demonstrated that vascular endothelial growth factor (VEGF) promotes endothelial migration through mtROS in cultured human umbilical vein endothelial cells [130]. Endothelial migration is critical in a variety of physiological conditions including wound healing and vascular repair. VEGF increases mitochondrial metabolism and mtROS production. Furthermore, mitochondria-targeted antioxidant prevents VEGF-induced endothelial migration. Secondly, mtROS contribute to endothelium-dependent vasodilation [131]. The endothelium regulates vascular homeostasis in response to shear stress by synthesizing vasodilators such as NO. Using ESR and histochemofluorescence methods, it has been shown that shear flow increases the production of ROS in human coronary resistance arteries, which can be blocked by mitochondrial complex I inhibitor rotenone. Moreover, complex I and complex III inhibitors, but not NADPH oxidase inhibitors, markedly blocked flow-induced dilation. Notably, ROS formation in response to flow after endothelial denudation is significantly decreased, suggesting an important role of endothelium in flow-induced mtROS formation. Thirdly, mtROS are also of importance in hypoxia-induced adaptation response in ECs. At low O_2_ concentrations, mitochondria of HUVECs (human umbilical vein endothelial cells) have been shown to generate ROS for activation of enzymes such as AMPK (AMP-activated protein kinase) because: 1) AMPK activation coincides with the hypoxia condition at which ROS is produced; 2) antioxidants can rescue hypoxia-induced AMPK activation; and 3) AMPK activation does not occur in ρ^0^ HUVECs devoid of mitochondria. Under the challenge of cardiovascular risk factors, however, excessive mtROS are produced in endothelial mitochondria. Vascular stressors, as diverse as oxidized lipids, high glucose, and angiotensin II, can all induce mtROS in ECs. (Figure 5, Table 3).

**Table 3:** Identified pathologic stressors that induce mtROS in endothelial cells (ECs)

**Cell type**	**Treatment**	**Dose**	**Time**	**PMID**
Bovine aortic ECs	Oxidized LDL	200 μg/ml	30 min	15805232
Bovine aortic ECs	Electrophilic lipids	4 μM	4 h	16387790
Human umbilical vein ECs	LysoPC	5 μmol/L	60 min	16651638
Porcine aortic ECs	Glycated LDL	100 μg/ml	2 h	20036735
Bovine aortic ECs	Glucose	30 mM	2 h	10783895
Human aorta ECs	Glucose	25 mM	7 d	12600878
bEnd3 microvascular ECs	Glucose	40 mM	7 d	21808008
Human umbilical vein ECs	Low Glucose	≤ 80 mg/dL	5 min	22207730
Bovine aortic ECs	Angiotensin II	200 nmol/L	4 h	18096818
Human aortic ECs	Angiotensin II	200 nmol/L	4 h	20448215
Human aortic ECs	Homocysteine	150 μM	24 h	21672628
Human umbilical vein ECs	Hypoxia	14 mmHg	30 min	11950692
Human umbilical vein ECs	Hypoxia	0 mmHg	24 h	12165534
Porcine aortic ECs	Hypoxia	0 mmHg	1 h	12690038
Murine pulmonary ECs	Thrombin	1 mU/mL	100 s	17724077
Human umbilical vein ECs	PAR1-AP	50 μmol/L	1 h	18983479
Human umbilical vein ECs	TNF-α	1 ng/mL	1 h	11415943
Murine pulmonary ECs	TNF-α	1 ng/mL	10 min	21519143
Bovine aortic ECs	Leptin	10 ng/mL	45 min	11342529
Human coronary artery ECs	Resistin	40/80 ng/mL	24 h	20435848
Human umbilical vein ECs	VEGF	50 ng/mL	5 min	21653897

PMID: PubMed ID.

Oxidized low density lipoprotein (oxLDL). Substantial evidence suggests that the retention and oxidative modification of LDL and subsequently activation of ECs initiates atherosclerotic lesion formation [132]. OxLDL triggers the expression of adhesion molecules and secretion of chemokines by ECs, which drive immune cell infiltration. In this sense, oxLDL has been shown to induce mtROS in ECs in vitro [102,133,134]. Using confocal microscopy, it has been demonstrated that a significant proportion of oxLDL-induced cellular ROS are co-localized to mitochondria. Moreover, ECs that are deficient in functional mitochondria show a substantial decrease in cellular ROS formation stimulated by oxLDL [102]. The precise mechanisms whereby oxLDL induces excessive mtROS generation in ECs remains poorly defined. It has been suggested that oxLDL significantly reduces oxygen consumption and enzyme activity in the mitochondrial ETC [133]. OxLDL also increases mitochondrial membrane potential and reduces SOD2 protein levels [134]. Moreover, c-Jun N-terminal kinases (JNK, one of the MAPKs) small interference RNA (siJNK) reduces oxLDL induced mtROS production substantially. OxLDL contains highly heterogeneous mixtures of biologically active substances [135]. Lysophosphatidylcholine (LysoPC, one of the active component derived from oxLDL) accounts for nearly 50% of the phosphatidylcholine equivalents in oxLDL and is considered as a critical factor that contributes to the proatherogenic activity of oxLDL [136]. As such, one study suggests that ROS production by lysoPC occurs predominantly in the mitochondria and is associated with an increase in mitochondrial Ca^2+^ uptake [104].

Hyperglycemia-high glucose. Hyperglycemia is a key cardiovascular risk factor for patients with type 2 diabetes [137]. High glucose is the first identified pathogenic stressor that induces mtROS in ECs [111]. High glucose-induced DCF fluorescence in bovine aortic ECs is prevented by several factors including an inhibitor of the ETC complex, an uncoupler of oxidative phosphorylation, uncoupling protein-1 and SOD2 [111]. Furthermore, normalizing levels of mtROS after treatment of cells with each of these agents prevents high glucose-induced activation of protein kinase C, formation of advanced glycation end-products, sorbitol accumulation and NFκB activation [111]. Later on, other studies report similar results [112,138]. One study shows that inhibition of ROS production by uncoupling of the ETC significantly reduces high glucose-mediated induction of IL-8 expression in human aortic ECs [112]. Another study links high glucose-dependent mtROS to increased consumption of H_2_S [138]. Interestingly, traditional pharmacological drugs including anti-inflammatory Sirt1 activator resveratrol, anti-inflammatory/anti-cancer drug cannabidiol, and hypolipidemic drug simvastatin have been shown to prevent high glucose-induced mtROS [139-141]. The measurement of MitoSOX fluorescence shows that resveratrol attenuates high glucose-induced mtROS production in human coronary arterial ECs. The authors propose that resveratrol, via a pathway that involves the upregulation of antioxidant defense mechanism, attenuates mtROS production [139]. Another paper shows that high glucose markedly increases mtROS, NF-κB activation, upregulation of iNOS, and EC adhesion molecules intercellular adhesion molecule-1 (ICAM-1) and vascular cell adhesion molecule-1 (VCAM-1), transendothelial migration of monocytes, and monocyte-endothelial adhesion in human coronary artery ECs. Remarkably, all the above mentioned effects induced by high glucose are attenuated by cannabidiol pretreatment [140]. Similarly, Simvastatin decreases high glucose-induced mtROS in bovine retinal capillary ECs and exerts protective effects against early retinal vascular damage in diabetic rats [141]. A recent study proposes that acute exposure to low glucose also increases mtROS production in human umbilical vein ECs [142]. Interestingly, anti-diabetic drug Metformin can reverse low glucose-induced endothelial dysfunction through inhibiting excessive mtROS production.

Angiotensin II. Ang II is another pathogenic stressor that mediates endothelial dysfunction and promotes vascular inflammation and atherogenesis [143]. Ang II treatment of bovine aortic ECs is shown to significantly increase mtROS production detected by ESR. This effect is associated with decreased endothelial NO availability [74]. Later on, the same group confirms this result using MitoSOX fluorescent probe. Interestingly, supplementation of human aorta ECs with the mitochondria-targeted antioxidant mitoTEMPO abolishes the MitoSOX signal after Ang II stimulation [78]. In addition, mitoTEMPO also prevents the loss of endothelial NO caused by Ang II both in cultured ECs and intact mice. Furthermore, treatment of hypertensive mice with mitoTEMPO after onset of Ang II-induced hypertension significantly reduces blood pressure and substantially improves endothelium-dependent vasodilation.

### Macrophages (MΦ) and dendritic cells (DCs)

The mononuclear phagocytic system consists of monocytes, MΦ, and DCs [144]. These cells all originate from the same haematopoietic precursors located in the bone marrow and their main functions are phagocytosis, cytokine secretion and antigen presentation. MΦ and DCs precursors are released into the circulation as monocytes, and within several days they exit the circulation through the endothelium into body tissues and differentiate into mature MΦ and DCs.

The phagocytic response of MΦ involves the production of ROS via NADPH-oxidase-dependent respiratory burst. However, recent studies have suggested that mtROS also have an important role in MΦ innate immune response [145]. Activation of a subset of Toll-like receptors (TLR1, TLR2, and TLR4) results in the recruitment of mitochondria to MΦ phagosomes and increased production of mtROS. This augmentation of mtROS production involves the engagement of a TLR signaling adaptor, tumor necrosis factor receptor-associated factor 6 (TRAF6), and the protein evolutionarily conserved signaling intermediate in Toll pathways) in the mitochondria. ECSIT is implicated in mitochondrial ETC assembly. Interaction between the two molecules then leads to ECSIT ubiquitination and enrichment around mitochondria, resulting in increased mtROS production. Additionally, scavenging mtROS in MΦ by expressing catalase in the mitochondria results in defective bacterial killing, confirming the important role of mtROS in MΦ bactericidal activity.

DCs are potent antigen-presenting cells, capable of inducing T and B responses as well as immune tolerance. Compare to its precursor monocytes, DCs exhibit significantly larger number of mitochondria and higher endogenous respiratory activity [146]. Complex I inhibitor rotenone prevents the increase in mitochondrial number as well as DC differentiation. Moreover, rotenone and catalase treatment both inhibit growth factor-induced mtROS in DCs, indicating that the differentiation of DC can be regulated by mtROS in DCs.

### Smooth muscle cells (SMCs)

Vascular SMCs migrate and proliferate when they are stimulated by cytokines and fibrogenic factors [147]. MtROS play an important role in this process as demonstrated by the finding that they mediate the activation of Akt/NFκB signaling pathway in response to 4-hydrocynonenal (4-HNE) stimulation in vascular SMCs [148]. In addition, mtROS in SMCs also play a role in cold-induced constriction of cutaneous arteries [149]. Cold constricts cutaneous arteries by selectively increasing the activity of α_2_-adrenoceptors (α_2_-ARs). Complex I inhibitor rotenone can abolish cold-induced increase in α_2_-ARs activity and dramatically inhibits cold-induced constriction response as well. In the atherosclerotic disease setting, thickened plaques render vascular SMCs prone to be hypoxic because of poor perfusion. Complex I inhibitor rotenone again abolishes hypoxia-induced HIF-1α protein expression and ROS generation, suggesting a critical role of mtROS in this pathological condition [150].

### MtROS and cancers

Mitochondria lie at the center of the metabolic theory of cancer [151]. Normally, differentiated cells primarily rely on mitochondrial respiration to produce ATP in the presence of oxygen (generating 36 mol. ATP/mol. glucose). Only under limited oxygen availability will healthy cells rely on anaerobic glycolysis as their energy source (generating 2 mol. ATP/mol. glucose). However, most cancer cells adopt the way of “aerobic glycolysis” for energy production [151]. This phenomenon, also known as the Warburg effect [152], is initially confusing to scientists as it is highly energy inefficient. However, it was later found that cancer cells have other important metabolic requirements that extend beyond the generation of ATP. On one aspect, cancer cells are particularly challenged in dealing with oxidative stress [153]. In highly proliferative cancer cells, the presence of oncogenic mutations promotes aberrant metabolism and protein translation, resulting in increased rates of ROS production. It was found that the Warburg effect is beneficial to transformed cells by upregulating antioxidant systems to counteract the accumulation of ROS. One key glycolytic enzyme – pyruvate kinase, plays an important role in this process: tumor cells express exclusively the embryonic M2 isoform of this enzyme (PKM2) [154] and an increase in intracellular ROS can inhibit PKM2 by oxidation of one reactive cysteine residue in this enzyme [155]. This inhibition of PKM2 then leads to the production of reducing equivalents to detoxify ROS by diverting glucose metabolism into pentose phosphate pathway [155]. By doing this, the regulatory properties of PKM2 provide cancer cells the protection against excessive mtROS production commonly seen in cancer [156].

### MtROS and hypertension

Hypertension is associated with increased ROS production in several key target organs, including the vasculature, the kidney, and the central nervous system, which all contribute to the regulation of blood pressure [157]. Ang II, the hormone commonly implicated in hypertension, is shown to increase ROS production in these sites. A key role of NADPH oxidase in this process has been demonstrated both in vitro and in vivo [158]. However, later studies indicates that Ang II activation of NADPH oxidase further leads to mitochondrial dysfunction and increased mtROS production [74]. Importantly, mice transgenic for Trx2, the mitochondrial antioxidant enzyme, are shown to resist the development of Ang II-induced hypertension and endothelial dysfunction [159]. Moreover, Ang II-induced hypertension is also significantly attenuated by either overexpressing SOD2 or treatment with mitoTEMPO [78]. These studies strongly demonstrate the potential of antioxidant strategies specifically targeting mitochondria as therapy in hypertension and possibly other diseases.

### MtROS and atherosclerosis

Multiple lines of in vivo experimental data indicate that excessive mtROS within vasculature promote the development of atherosclerosis. ApoE^−/−^ mice that are deficient in SOD2, a mitochondria-specific antioxidant enzyme, exhibit accelerated atherogenesis at arterial branching points [160]. SOD2 is also shown to protect against endothelial dysfunction in carotid artery of ApoE^−/−^ mice [20]. Notably, transgenic overexpression of Trx2, another mitochondrial antioxidant enzyme, improves endothelial function and reduces atherosclerotic lesions in ApoE^−/−^ mice in part by reducing oxidative stress and increasing NO bioavailability [27]. There is still limited knowledge of the involvement of mtROS in human atherogenesis; however, epidemiologic data suggest that genetic nucleotide polymorphisms leading to reduced SOD2 function are associated with increased atherosclerotic risk [161]. In addition, there is significantly increased mtDNA damage in atherosclerotic human arterial specimens compared to that of normal human arterial tissue [160]. Indeed, increased mtDNA damage is also a shared phenotype of multiple diseases including neurological degenerative disease [162] and cancer [163]. As mtDNA contains genes that encode critical structural subunits for three of the four protein complexes of the ETC (complex I, III, and IV) (Table 1) [164], mtDNA damage will lead to increases in mtROS generation and the extent of mtDNA damage is an index of the levels of mtROS.

### MtROS and other inflammatory diseases

It has long been recognized that ROS are implicated in many inflammatory diseases other than atherosclerosis, including multiple sclerosis [165], rheumatoid arthritis [166], thyroiditis [167], and type 1 diabetes [168]. Very recently, however, it has been identified that mitochondria-derived ROS rather than NADPH oxidase-derived ROS promote the production of proinflammatory cytokines in TNFR1-associated periodic syndrome (TRAPS) [6]. TRAPS is an autoinflammatory disorder associated with enhanced innate immune responsiveness. It is caused by mutations of the gene encoding type 1 TNF receptor (TNFR1), which leads to aberrant activation of MAPKs [169]. It has been found that mtROS, mitochondrial oxidative capacity, and Δψ_m_ are all increased in human patient cells and mouse cells harboring TRAPS-related TNFR1 mutations [6]. Moreover, scavenging mtROS using MitoQ abolishes inflammatory cytokine production after LPS stimulation in these cells, highlighting the potential of targeting mtROS as novel therapy for TRAPS and other inflammatory diseases.

## Conclusions

Oxidative stress has long been recognized as a major player in the development of atherosclerosis and other inflammatory diseases. This has led to the enthusiastic use of antioxidants in the prevention and treatment of diseases. However, the results of randomized clinical trials of Vitamin C and Vitamin E have been disappointing [170-173]. If indeed oxidative stress is so crucial in the development and manifestations of atherosclerosis-related diseases including myocardial infarction and stroke, why have so many clinical trials failed to demonstrate its therapeutic efficacy? One possibility may be related to the fact that only a small proportion of known antioxidants in vivo are actually located in the mitochondria. Given the fact that mtROS directly drive proinflammatory cytokine production, we can speculate that specific targeting of mtROS may result in better outcome in combating chronic inflammatory diseases such as atherosclerosis. However, in order to translate this knowledge from benchtop to bedside, future studies that fully characterize the biochemistry of mtROS and the specific role mtROS plays in these inflammatory diseases need to be performed. We hope that our review will encourage investigators to enter this important field of research and to accelerate the pace of translational medicine and therapeutics.

## Abbreviations

4HNE: 4-hydrocynonenal (4-HNE); ADP: adenosine diphosphate; AIM2: Absent in melanoma 2; AMPK: AMP-activated protein kinase; Ang II: Angiotensin II; APAF: Apoptosis activating factor; ApoE: Apolipoprotein E; ATG4: Autophagy-specific gene 4; ATP: Adenosine triphosphate; BH4: Tetrahydrobiopterin; CaM: Calmodulin; COX: Cytochrome c oxidase subunit; CPT1: Carnitine palmitoyl transferase I; CREB: cAMP-responsive transcription factor; CVD: Cardiovascular disease; DCF: Dichlorodihydrofluorescein; DCs: Dendritic cells; DHE: Dihydroethidium; ECs: Endothelial cells; ECSIT: Evolutionarily conserved signaling intermediate in Toll pathways; eNOS: Endothelial NOS; ER: Endoplasmic reticulum; ERRα: Estrogen-related receptor alpha; ESR: Electron spin resonance; ETC: Electron transport chain; FADH2: Flavin adenine dinucleotide; FAO: Fatty acid oxidation; FCCP: carbonyl cyanide p-(tri-fluromethoxy)phenyl-hydrazone; GPx: Glutathione peroxidase; GR: gLutathione reductase; GSH: Glutathione; GSSG: Oxidized glutathione; HIF-1α: Hypoxia-inducible factor 1α;HPLC: High-performance liquid chromatography; hROS: Highly reactive oxygen species; HUVECs: Human umbilical vein endothelial cells; ICAM-1: Intercellular adhesion molecule-1; IFN-γ: Interferon-γ; IL: Interleukin; iNOS: Inducible NOS; IRF: Interferon regulatory transcription factor; JNK: c-Jun N-terminal kinases; LPS: Lipopolysaccharide; LysoPC: Lysophosphatidylcholine; MAPK: Mitogen-activated protein kinases; MEF2D: Myocyte enhancer factor-2D; mitoTFs: Mitochondrial TFs; MPOs: Myeloperoxidases; mtDNA: Mitochondrial DNA; mtROS: Mitochondrial ROS; MΦ: Macrophages; NADH: nicotine adenine dinucleotide; NADPH: Nicotinamide adenine dinucleotide phosphate; NDUFA4L2: NADH dehydrogenase [ubiquinone] 1 alpha subcomplex, 4-like 2; NF-κB: Nuclear factor kappa B; NLRC4: NLR family, CARD-containing 4; NLRP3: NOD-like receptor family, pyrin domain containing 3; NLRP6: NOD-like receptor family, pyrin domain containing 6; nNOS: Neuronal NOS; NOS: Nitric oxide synthase; NOX: NADPH oxidase; oxLDL: Oxidized low density lipoprotein; OXPHOS: Oxidative phosphorylation; PGC1α: Peroxisome-proliferator-activated receptor-γ coactivator 1α; PKA: Protein kinase A; PKM2: Pyruvate kinase M2 isozyme; PPAR: Peroxisome proliferator-activated receptor; RLRs: RIG-I-like receptors; ROS: Reactive oxygen species; SMCs: Smooth muscle cells; SNARE: N-ethylmaleimide-sensitive factor attachment protein receptor; SOD: Superoxide dismutase; SRC: Spare respiratory capacity; STAT: Signal transducer and activator of transcription; TFs: Transcription factors; TLR: Toll-like receptors; TNF: Tumor necrosis factor; TNFR1: Type 1 TNF receptor; TPP+: Triphenylphosphonium; TRAF6: Tumor necrosis factor receptor-associated factor 6; Trx: Thioredoxin; UCPs: Uncoupling proteins; VCAM-1: Vascular cell adhesion molecule-1; XO: Xanthine oxidase; α2-ARs: α 2-adrenoceptors; Δψm: Mitochondrial membrane potential

## Competing interests

The authors declare that they have no competing interests.

## Authors’ contribution

XL carried out the primary literature search and drafted the manuscript. PF, JM, and ETC provided material input and helped revising the manuscript. HW and XFY conceived the study and provided field expertise. All authors read and approved the final manuscript.

## References

[B1] Winterbourn CC Reconciling the chemistry and biology of reactive oxygen species Nat Chem Biol 2008 4 278 286 18421291 10.1038/nchembio.85

[B2] Block K Gorin Y Aiding and abetting roles of NOX oxidases in cellular transformation Nat Rev Cancer 2012 12 627 637 22918415 10.1038/nrc3339 PMC3711509

[B3] West AP Shadel GS Ghosh S Mitochondria in innate immune responses Nat Rev Immunol 2011 11 389 402 21597473 10.1038/nri2975 PMC4281487

[B4] Tal MC Sasai M Lee HK Yordy B Shadel GS Iwasaki A Absence of autophagy results in reactive oxygen species-dependent amplification of RLR signaling Proc Natl Acad Sci U S A 2009 106 2770 2775 19196953 10.1073/pnas.0807694106 PMC2650341

[B5] Zhou R Yazdi AS Menu P Tschopp J A role for mitochondria in NLRP3 inflammasome activation Nature 2010 469 221 225 21124315 10.1038/nature09663

[B6] Bulua AC Simon A Maddipati R Pelletier M Park H Kim KY Sack MN Kastner DL Siegel RM Mitochondrial reactive oxygen species promote production of proinflammatory cytokines and are elevated in TNFR1-associated periodic syndrome (TRAPS) J Exp Med 2011 208 519 533 21282379 10.1084/jem.20102049 PMC3058571

[B7] Nakashima Y Chen YX Kinukawa N Sueishi K Distributions of diffuse intimal thickening in human arteries: preferential expression in atherosclerosis-prone arteries from an early age Virchows Arch 2002 441 279 288 12242525 10.1007/s00428-002-0605-1

[B8] Strong JP Malcom GT McMahan CA Tracy RE Newman WP 3rd Herderick EE Cornhill JF Prevalence and extent of atherosclerosis in adolescents and young adults: implications for prevention from the Pathobiological Determinants of Atherosclerosis in Youth Study Jama 1999 281 727 735 10052443 10.1001/jama.281.8.727

[B9] Ross R Atherosclerosis–an inflammatory disease N Engl J Med 1999 340 115 126 9887164 10.1056/NEJM199901143400207

[B10] Tedgui A Mallat Z Cytokines in atherosclerosis: pathogenic and regulatory pathways Physiol Rev 2006 86 515 581 16601268 10.1152/physrev.00024.2005

[B11] Madamanchi NR Runge MS Mitochondrial dysfunction in atherosclerosis Circ Res 2007 100 460 473 17332437 10.1161/01.RES.0000258450.44413.96

[B12] Zhang DX Gutterman DD Mitochondrial reactive oxygen species-mediated signaling in endothelial cells Am J Physiol Heart Circ Physiol 2007 292 H2023 H2031 17237240 10.1152/ajpheart.01283.2006

[B13] Handy DE Loscalzo J Redox regulation of mitochondrial function Antioxid Redox Signal 2012 16 1323 1367 22146081 10.1089/ars.2011.4123 PMC3324814

[B14] Tschopp J Mitochondria: Sovereign of inflammation? Eur J Immunol 2011 41 1196 1202 21469137 10.1002/eji.201141436

[B15] Aon MA Cortassa S O'Rourke B Redox-optimized ROS balance: a unifying hypothesis Biochim Biophys Acta 2010 1797 865 877 20175987 10.1016/j.bbabio.2010.02.016 PMC2891851

[B16] Han D Canali R Rettori D Kaplowitz N Effect of glutathione depletion on sites and topology of superoxide and hydrogen peroxide production in mitochondria Mol Pharmacol 2003 64 1136 1144 14573763 10.1124/mol.64.5.1136

[B17] Cadenas E Davies KJ Mitochondrial free radical generation, oxidative stress, and aging Free Radic Biol Med 2000 29 222 230 11035250 10.1016/s0891-5849(00)00317-8

[B18] Okado-Matsumoto A Fridovich I Subcellular distribution of superoxide dismutases (SOD) in rat liver: Cu, Zn-SOD in mitochondria J Biol Chem 2001 276 38388 38393 11507097 10.1074/jbc.M105395200

[B19] Li Y Huang TT Carlson EJ Melov S Ursell PC Olson JL Noble LJ Yoshimura MP Berger C Chan PH Dilated cardiomyopathy and neonatal lethality in mutant mice lacking manganese superoxide dismutase Nat Genet 1995 11 376 381 7493016 10.1038/ng1295-376

[B20] Ohashi M Runge MS Faraci FM Heistad DD MnSOD deficiency increases endothelial dysfunction in ApoE-deficient mice Arterioscler Thromb Vasc Biol 2006 26 2331 2336 16873728 10.1161/01.ATV.0000238347.77590.c9

[B21] Radi R Turrens JF Chang LY Bush KM Crapo JD Freeman BA Detection of catalase in rat heart mitochondria J Biol Chem 1991 266 22028 22034 1657986

[B22] Yang H Roberts LJ Shi MJ Zhou LC Ballard BR Richardson A Guo ZM Retardation of atherosclerosis by overexpression of catalase or both Cu/Zn-superoxide dismutase and catalase in mice lacking apolipoprotein E Circ Res 2004 95 1075 1081 15528470 10.1161/01.RES.0000149564.49410.0d

[B23] Schriner SE Linford NJ Martin GM Treuting P Ogburn CE Emond M Coskun PE Ladiges W Wolf N Van Remmen H Extension of murine life span by overexpression of catalase targeted to mitochondria Science 2005 308 1909 1911 15879174 10.1126/science.1106653

[B24] Torzewski M Ochsenhirt V Kleschyov AL Oelze M Daiber A Li H Rossmann H Tsimikas S Reifenberg K Cheng F Deficiency of glutathione peroxidase-1 accelerates the progression of atherosclerosis in apolipoprotein E-deficient mice Arterioscler Thromb Vasc Biol 2007 27 850 857 17255533 10.1161/01.ATV.0000258809.47285.07

[B25] Rhee SG Chae HZ Kim K Peroxiredoxins: a historical overview and speculative preview of novel mechanisms and emerging concepts in cell signaling Free Radic Biol Med 2005 38 1543 1552 15917183 10.1016/j.freeradbiomed.2005.02.026

[B26] Matsushima S Ide T Yamato M Matsusaka H Hattori F Ikeuchi M Kubota T Sunagawa K Hasegawa Y Kurihara T Overexpression of mitochondrial peroxiredoxin-3 prevents left ventricular remodeling and failure after myocardial infarction in mice Circulation 2006 113 1779 1786 16585391 10.1161/CIRCULATIONAHA.105.582239

[B27] Zhang H Luo Y Zhang W He Y Dai S Zhang R Huang Y Bernatchez P Giordano FJ Shadel G Endothelial-specific expression of mitochondrial thioredoxin improves endothelial cell function and reduces atherosclerotic lesions Am J Pathol 2007 170 1108 1120 17322393 10.2353/ajpath.2007.060960 PMC1864879

[B28] He M Cai J Go YM Johnson JM Martin WD Hansen JM Jones DP Identification of thioredoxin-2 as a regulator of the mitochondrial permeability transition Toxicol Sci 2008 105 44 50 18550601 10.1093/toxsci/kfn116 PMC2734306

[B29] Sokol RJ McKim JM Jr Goff MC Ruyle SZ Devereaux MW Han D Packer L Everson G Vitamin E reduces oxidant injury to mitochondria and the hepatotoxicity of taurochenodeoxycholic acid in the rat Gastroenterology 1998 114 164 174 9428230 10.1016/s0016-5085(98)70644-4

[B30] Gilgun-Sherki Y Melamed E Offen D Oxidative stress induced-neurodegenerative diseases: the need for antioxidants that penetrate the blood brain barrier Neuropharmacology 2001 40 959 975 11406187 10.1016/s0028-3908(01)00019-3

[B31] Murphy MP Smith RA Targeting antioxidants to mitochondria by conjugation to lipophilic cations Annu Rev Pharmacol Toxicol 2007 47 629 656 17014364 10.1146/annurev.pharmtox.47.120505.105110

[B32] Smith RA Porteous CM Coulter CV Murphy MP Selective targeting of an antioxidant to mitochondria Eur J Biochem 1999 263 709 716 10469134 10.1046/j.1432-1327.1999.00543.x

[B33] Davidson SM Endothelial mitochondria and heart disease Cardiovasc Res 2010 88 58 66 20558442 10.1093/cvr/cvq195

[B34] Kelso GF Porteous CM Coulter CV Hughes G Porteous WK Ledgerwood EC Smith RA Murphy MP Selective targeting of a redox-active ubiquinone to mitochondria within cells: antioxidant and antiapoptotic properties J Biol Chem 2001 276 4588 4596 11092892 10.1074/jbc.M009093200

[B35] Ballinger SW Mitochondrial dysfunction in cardiovascular disease Free Radic Biol Med 2005 38 1278 1295 15855047 10.1016/j.freeradbiomed.2005.02.014

[B36] Toime LJ Brand MD Uncoupling protein-3 lowers reactive oxygen species production in isolated mitochondria Free Radic Biol Med 2010 49 606 611 20493945 10.1016/j.freeradbiomed.2010.05.010 PMC2903626

[B37] Brennan JP Southworth R Medina RA Davidson SM Duchen MR Shattock MJ Mitochondrial uncoupling, with low concentration FCCP, induces ROS-dependent cardioprotection independent of KATP channel activation Cardiovasc Res 2006 72 313 321 16950237 10.1016/j.cardiores.2006.07.019

[B38] Lee KU Lee IK Han J Song DK Kim YM Song HS Kim HS Lee WJ Koh EH Song KH Effects of recombinant adenovirus-mediated uncoupling protein 2 overexpression on endothelial function and apoptosis Circ Res 2005 96 1200 1207 15905464 10.1161/01.RES.0000170075.73039.5b

[B39] Chance B Williams GR Respiratory enzymes in oxidative phosphorylation. III. The steady state J Biol Chem 1955 217 409 427 13271404

[B40] Andriantsitohaina R Duluc L Garcia-Rodriguez JC Gil-del Valle L, Guevara-Garcia M, Simard G, Soleti R, Su DF, Velasquez-Perez L, Wilson JX, Laher I: Systems biology of antioxidants Clin Sci (Lond) 2012 123 173 192 22494160 10.1042/CS20110643

[B41] Stuehr DJ Structure-function aspects in the nitric oxide synthases Annu Rev Pharmacol Toxicol 1997 37 339 359 9131257 10.1146/annurev.pharmtox.37.1.339

[B42] Ghafourifar P Richter C Nitric oxide synthase activity in mitochondria FEBS Lett 1997 418 291 296 9428730 10.1016/s0014-5793(97)01397-5

[B43] Shiva S Mitochondria as metabolizers and targets of nitrite Nitric Oxide 2010 22 64 74 19788924 10.1016/j.niox.2009.09.002 PMC2819587

[B44] Feissner RF Skalska J Gaum WE Sheu SS Crosstalk signaling between mitochondrial Ca2+ and ROS Front Biosci 2009 14 1197 1218 19273125 10.2741/3303 PMC2683671

[B45] Peng TI Jou MJ Oxidative stress caused by mitochondrial calcium overload Ann N Y Acad Sci 2010 1201 183 188 20649555 10.1111/j.1749-6632.2010.05634.x

[B46] Turrens JF Freeman BA Levitt JG Crapo JD The effect of hyperoxia on superoxide production by lung submitochondrial particles Arch Biochem Biophys 1982 217 401 410 6291460 10.1016/0003-9861(82)90518-5

[B47] Guzy RD Hoyos B Robin E Chen H Liu L Mansfield KD Simon MC Hammerling U Schumacker PT Mitochondrial complex III is required for hypoxia-induced ROS production and cellular oxygen sensing Cell Metab 2005 1 401 408 16054089 10.1016/j.cmet.2005.05.001

[B48] McLeod CJ Aziz A Hoyt RF Jr McCoy JP Jr Sack MN Uncoupling proteins 2 and 3 function in concert to augment tolerance to cardiac ischemia J Biol Chem 2005 280 33470 33476 16079144 10.1074/jbc.M505258200

[B49] St-Pierre J Drori S Uldry M Silvaggi JM Rhee J Jager S Handschin C Zheng K Lin J Yang W Suppression of reactive oxygen species and neurodegeneration by the PGC-1 transcriptional coactivators Cell 2006 127 397 408 17055439 10.1016/j.cell.2006.09.024

[B50] Okamoto K Kondo-Okamoto N Mitochondria and autophagy: critical interplay between the two homeostats Biochim Biophys Acta 1820 2012 595 600 10.1016/j.bbagen.2011.08.001 21846491

[B51] Archer SL Gomberg-Maitland M Maitland ML Rich S Garcia JG Weir EK Mitochondrial metabolism, redox signaling, and fusion: a mitochondria-ROS-HIF-1alpha-Kv1.5 O2-sensing pathway at the intersection of pulmonary hypertension and cancer Am J Physiol Heart Circ Physiol 2008 294 H570 H578 18083891 10.1152/ajpheart.01324.2007

[B52] Pangare M Makino A Mitochondrial function in vascular endothelial cell in diabetes J Smooth Muscle Res 2012 48 1 26 22504486 10.1540/jsmr.48.1 PMC3655204

[B53] Szczepanek K Lesnefsky EJ Larner AC Multi-tasking: nuclear transcription factors with novel roles in the mitochondria Trends Cell Biol 2012 22 429 437 22705015 10.1016/j.tcb.2012.05.001 PMC3516366

[B54] Wegrzyn J Potla R Chwae YJ Sepuri NB Zhang Q Koeck T Derecka M Szczepanek K Szelag M Gornicka A Function of mitochondrial Stat3 in cellular respiration Science 2009 323 793 797 19131594 10.1126/science.1164551 PMC2758306

[B55] Semenza GL Hypoxia-inducible factor 1: regulator of mitochondrial metabolism and mediator of ischemic preconditioning Biochim Biophys Acta 1813 2011 1263 1268 10.1016/j.bbamcr.2010.08.006 PMC3010308 20732359

[B56] Sauve AA Sirtuin chemical mechanisms Biochim Biophys Acta 1804 2010 1591 1603 10.1016/j.bbapap.2010.01.021 PMC2886189 20132909

[B57] Gertz M Steegborn C Function and regulation of the mitochondrial sirtuin isoform Sirt5 in Mammalia Biochim Biophys Acta 1804 2009 1658 1665 10.1016/j.bbapap.2009.09.011 19766741

[B58] Zhang QJ Wang Z Chen HZ Zhou S Zheng W Liu G Wei YS Cai H Liu DP Liang CC Endothelium-specific overexpression of class III deacetylase SIRT1 decreases atherosclerosis in apolipoprotein E-deficient mice Cardiovasc Res 2008 80 191 199 18689793 10.1093/cvr/cvn224 PMC3657473

[B59] Orimo M Minamino T Miyauchi H Tateno K Okada S Moriya J Komuro I Protective role of SIRT1 in diabetic vascular dysfunction Arterioscler Thromb Vasc Biol 2009 29 889 894 19286634 10.1161/ATVBAHA.109.185694

[B60] Howitz KT Bitterman KJ Cohen HY Lamming DW Lavu S Wood JG Zipkin RE Chung P Kisielewski A Zhang LL Small molecule activators of sirtuins extend Saccharomyces cerevisiae lifespan Nature 2003 425 191 196 12939617 10.1038/nature01960

[B61] Khanduja KL Bhardwaj A Kaushik G: Resveratrol inhibits N-nitrosodiethylamine-induced ornithine decarboxylase and cyclooxygenase in mice J Nutr Sci Vitaminol (Tokyo) 2004 50 61 65 15228220

[B62] Baur JA Pearson KJ Price NL Jamieson HA Lerin C Kalra A Prabhu VV Allard JS Lopez-Lluch G Lewis K Resveratrol improves health and survival of mice on a high-calorie diet Nature 2006 444 337 342 17086191 10.1038/nature05354 PMC4990206

[B63] Docherty JJ Fu MM Hah JM Sweet TJ Faith SA Booth T Effect of resveratrol on herpes simplex virus vaginal infection in the mouse Antiviral Res 2005 67 155 162 16125258 10.1016/j.antiviral.2005.06.008

[B64] Jang M Cai L Udeani GO Slowing KV Thomas CF Beecher CW Fong HH Farnsworth NR Kinghorn AD Mehta RG Cancer chemopreventive activity of resveratrol, a natural product derived from grapes Science 1997 275 218 220 8985016 10.1126/science.275.5297.218

[B65] Chu LM Lassaletta AD Robich MP Sellke FW Resveratrol in the prevention and treatment of coronary artery disease Curr Atheroscler Rep 2011 13 439 446 21870059 10.1007/s11883-011-0202-3

[B66] Corda S Laplace C Vicaut E Duranteau J Rapid reactive oxygen species production by mitochondria in endothelial cells exposed to tumor necrosis factor-alpha is mediated by ceramide Am J Respir Cell Mol Biol 2001 24 762 768 11415943 10.1165/ajrcmb.24.6.4228

[B67] Hawkins BJ Solt LA Chowdhury I Kazi AS Abid MR Aird WC May MJ Foskett JK Madesh M G protein-coupled receptor Ca2 + −linked mitochondrial reactive oxygen species are essential for endothelial/leukocyte adherence Mol Cell Biol 2007 27 7582 7593 17724077 10.1128/MCB.00493-07 PMC2169045

[B68] Rowlands DJ Islam MN Das SR Huertas A Quadri SK Horiuchi K Inamdar N Emin MT Lindert J Ten VS Activation of TNFR1 ectodomain shedding by mitochondrial Ca2+ determines the severity of inflammation in mouse lung microvessels J Clin Invest 2011 121 1986 1999 21519143 10.1172/JCI43839 PMC3083796

[B69] Ouchi N Parker JL Lugus JJ Walsh K Adipokines in inflammation and metabolic disease Nat Rev Immunol 2011 11 85 97 21252989 10.1038/nri2921 PMC3518031

[B70] Yamagishi SI Edelstein D Du XL Kaneda Y Guzman M Brownlee M Leptin induces mitochondrial superoxide production and monocyte chemoattractant protein-1 expression in aortic endothelial cells by increasing fatty acid oxidation via protein kinase A J Biol Chem 2001 276 25096 25100 11342529 10.1074/jbc.M007383200

[B71] Chen C Jiang J Lu JM Chai H Wang X Lin PH Yao Q Resistin decreases expression of endothelial nitric oxide synthase through oxidative stress in human coronary artery endothelial cells Am J Physiol Heart Circ Physiol 2010 299 H193 H201 20435848 10.1152/ajpheart.00431.2009 PMC2904138

[B72] van der Windt GJ Everts B Chang CH Curtis JD Freitas TC Amiel E Pearce EJ Pearce EL Mitochondrial respiratory capacity is a critical regulator of CD8+ T cell memory development Immunity 2012 36 68 78 22206904 10.1016/j.immuni.2011.12.007 PMC3269311

[B73] Quinn LS Anderson BG Conner JD Wolden-Hanson T IL-15 Overexpression Promotes Endurance, Oxidative Energy Metabolism, and Muscle PPARdelta, SIRT1, PGC-1alpha, and PGC-1beta Expression in Male Mice Endocrinology 2012 10.1210/en.2012-1773 PMC3529369 23161867

[B74] Doughan AK Harrison DG Dikalov SI Molecular mechanisms of angiotensin II-mediated mitochondrial dysfunction: linking mitochondrial oxidative damage and vascular endothelial dysfunction Circ Res 2008 102 488 496 18096818 10.1161/CIRCRESAHA.107.162800

[B75] Baudry N Laemmel E Vicaut E In vivo reactive oxygen species production induced by ischemia in muscle arterioles of mice: involvement of xanthine oxidase and mitochondria Am J Physiol Heart Circ Physiol 2008 294 H821 H828 18055522 10.1152/ajpheart.00378.2007

[B76] Ceylan-Isik AF Guo KK Carlson EC Privratsky JR Liao SJ Cai L Chen AF Ren J Metallothionein abrogates GTP cyclohydrolase I inhibition-induced cardiac contractile and morphological defects: role of mitochondrial biogenesis Hypertension 2009 53 1023 1031 19398661 10.1161/HYPERTENSIONAHA.108.123422 PMC2782760

[B77] Hanna IR Taniyama Y Szocs K Rocic P Griendling KK NAD(P)H oxidase-derived reactive oxygen species as mediators of angiotensin II signaling Antioxid Redox Signal 2002 4 899 914 12573139 10.1089/152308602762197443

[B78] Dikalova AE Bikineyeva AT Budzyn K Nazarewicz RR McCann L Lewis W Harrison DG Dikalov SI Therapeutic targeting of mitochondrial superoxide in hypertension Circ Res 2010 107 106 116 20448215 10.1161/CIRCRESAHA.109.214601 PMC2901409

[B79] Giorgio M Migliaccio E Orsini F Paolucci D Moroni M Contursi C Pelliccia G Luzi L Minucci S Marcaccio M Electron transfer between cytochrome c and p66Shc generates reactive oxygen species that trigger mitochondrial apoptosis Cell 2005 122 221 233 16051147 10.1016/j.cell.2005.05.011

[B80] Gertz M Fischer F Wolters D Steegborn C Activation of the lifespan regulator p66Shc through reversible disulfide bond formation Proc Natl Acad Sci U S A 2008 105 5705 5709 18413607 10.1073/pnas.0800691105 PMC2311372

[B81] Shi Y Cosentino F Camici GG Akhmedov A Vanhoutte PM Tanner FC Luscher TF Oxidized low-density lipoprotein activates p66Shc via lectin-like oxidized low-density lipoprotein receptor-1, protein kinase C-beta, and c-Jun N-terminal kinase kinase in human endothelial cells Arterioscler Thromb Vasc Biol 2011 31 2090 2097 21817106 10.1161/ATVBAHA.111.229260

[B82] Napoli C Martin-Padura I de Nigris F Giorgio M Mansueto G Somma P Condorelli M Sica G De Rosa G Pelicci P Deletion of the p66Shc longevity gene reduces systemic and tissue oxidative stress, vascular cell apoptosis, and early atherogenesis in mice fed a high-fat diet Proc Natl Acad Sci U S A 2003 100 2112 2116 12571362 10.1073/pnas.0336359100 PMC149967

[B83] Finkel T Signal transduction by mitochondrial oxidants J Biol Chem 2012 287 4434 4440 21832045 10.1074/jbc.R111.271999 PMC3281633

[B84] Finkel T Signal transduction by reactive oxygen species J Cell Biol 2011 194 7 15 21746850 10.1083/jcb.201102095 PMC3135394

[B85] Fomenko DE Xing W Adair BM Thomas DJ Gladyshev VN High-throughput identification of catalytic redox-active cysteine residues Science 2007 315 387 389 17234949 10.1126/science.1133114

[B86] Weerapana E Wang C Simon GM Richter F Khare S Dillon MB Bachovchin DA Mowen K Baker D Cravatt BF Quantitative reactivity profiling predicts functional cysteines in proteomes Nature 2010 468 790 795 21085121 10.1038/nature09472 PMC3058684

[B87] Wang GL Jiang BH Rue EA Semenza GL Hypoxia-inducible factor 1 is a basic-helix-loop-helix-PAS heterodimer regulated by cellular O2 tension Proc Natl Acad Sci U S A 1995 92 5510 5514 7539918 10.1073/pnas.92.12.5510 PMC41725

[B88] Tello D Balsa E Acosta-Iborra B Fuertes-Yebra E Elorza A Ordonez A Corral-Escariz M Soro I Lopez-Bernardo E Perales-Clemente E Induction of the mitochondrial NDUFA4L2 protein by HIF-1alpha decreases oxygen consumption by inhibiting Complex I activity Cell Metab 2011 14 768 779 22100406 10.1016/j.cmet.2011.10.008

[B89] Gross O Thomas CJ Guarda G Tschopp J The inflammasome: an integrated view Immunol Rev 2011 243 136 151 21884173 10.1111/j.1600-065X.2011.01046.x

[B90] Strowig T Henao-Mejia J Elinav E Flavell R Inflammasomes in health and disease Nature 2012 481 278 286 22258606 10.1038/nature10759

[B91] Lamkanfi M Emerging inflammasome effector mechanisms Nat Rev Immunol 2011 11 213 220 21350580 10.1038/nri2936

[B92] Zitvogel L Kepp O Galluzzi L Kroemer G Inflammasomes in carcinogenesis and anticancer immune responses Nat Immunol 2012 13 343 351 22430787 10.1038/ni.2224

[B93] Kepp O Galluzzi L Zitvogel L Kroemer G Pyroptosis - a cell death modality of its kind? Eur J Immunol 2010 40 627 630 20201017 10.1002/eji.200940160

[B94] Bauernfeind F Bartok E Rieger A Franchi L Nunez G Hornung V Cutting edge: reactive oxygen species inhibitors block priming, but not activation, of the NLRP3 inflammasome J Immunol 2011 187 613 617 21677136 10.4049/jimmunol.1100613 PMC3131480

[B95] Yin Y Yan Y Jiang X Mai J Chen NC Wang H Yang XF Inflammasomes are differentially expressed in cardiovascular and other tissues Int J Immunopathol Pharmacol 2009 22 311 322 19505385 10.1177/039463200902200208 PMC2847797

[B96] Kamata H Honda S Maeda S Chang L Hirata H Karin M Reactive oxygen species promote TNFalpha-induced death and sustained JNK activation by inhibiting MAP kinase phosphatases Cell 2005 120 649 661 15766528 10.1016/j.cell.2004.12.041

[B97] Naik E Dixit VM Mitochondrial reactive oxygen species drive proinflammatory cytokine production J Exp Med 2011 208 417 420 21357740 10.1084/jem.20110367 PMC3058577

[B98] Pueyo ME Gonzalez W Nicoletti A Savoie F Arnal JF Michel JB Angiotensin II stimulates endothelial vascular cell adhesion molecule-1 via nuclear factor-kappaB activation induced by intracellular oxidative stress Arterioscler Thromb Vasc Biol 2000 20 645 651 10712386 10.1161/01.atv.20.3.645

[B99] Zamzami N Marchetti P Castedo M Decaudin D Macho A Hirsch T Susin SA Petit PX Mignotte B Kroemer G Sequential reduction of mitochondrial transmembrane potential and generation of reactive oxygen species in early programmed cell death J Exp Med 1995 182 367 377 7629499 10.1084/jem.182.2.367 PMC2192111

[B100] Riedl SJ Salvesen GS The apoptosome: signalling platform of cell death Nat Rev Mol Cell Biol 2007 8 405 413 17377525 10.1038/nrm2153

[B101] Scherz-Shouval R Shvets E Fass E Shorer H Gil L Elazar Z Reactive oxygen species are essential for autophagy and specifically regulate the activity of Atg4 Embo J 2007 26 1749 1760 17347651 10.1038/sj.emboj.7601623 PMC1847657

[B102] Zmijewski JW Moellering DR Le Goffe C Landar A Ramachandran A Darley-Usmar VM Oxidized LDL induces mitochondrially associated reactive oxygen/nitrogen species formation in endothelial cells Am J Physiol Heart Circ Physiol 2005 289 H852 H861 15805232 10.1152/ajpheart.00015.2005

[B103] Landar A Zmijewski JW Dickinson DA Le Goffe C Johnson MS Milne GL Zanoni G Vidari G Morrow JD Darley-Usmar VM Interaction of electrophilic lipid oxidation products with mitochondria in endothelial cells and formation of reactive oxygen species Am J Physiol Heart Circ Physiol 2006 290 H1777 H1787 16387790 10.1152/ajpheart.01087.2005

[B104] Watanabe N Zmijewski JW Takabe W Umezu-Goto M Le Goffe C Sekine A Landar A Watanabe A Aoki J Arai H Activation of mitogen-activated protein kinases by lysophosphatidylcholine-induced mitochondrial reactive oxygen species generation in endothelial cells Am J Pathol 2006 168 1737 1748 16651638 10.2353/ajpath.2006.050648 PMC1606607

[B105] Smith RA Hartley RC Murphy MP Mitochondria-targeted small molecule therapeutics and probes Antioxid Redox Signal 2011 15 3021 3038 21395490 10.1089/ars.2011.3969

[B106] Mukhopadhyay P Rajesh M Hasko G Hawkins BJ Madesh M Pacher P Simultaneous detection of apoptosis and mitochondrial superoxide production in live cells by flow cytometry and confocal microscopy Nat Protoc 2007 2 2295 2301 17853886 10.1038/nprot.2007.327 PMC2225540

[B107] Kalyanaraman B Darley-Usmar V Davies KJ Dennery PA Forman HJ Grisham MB Mann GE Moore K Roberts LJ 2nd Ischiropoulos H Measuring reactive oxygen and nitrogen species with fluorescent probes: challenges and limitations Free Radic Biol Med 2012 52 1 6 22027063 10.1016/j.freeradbiomed.2011.09.030 PMC3911769

[B108] Dickinson BC Chang CJ A targetable fluorescent probe for imaging hydrogen peroxide in the mitochondria of living cells J Am Chem Soc 2008 130 9638 9639 18605728 10.1021/ja802355u PMC2810491

[B109] Dickinson BC Srikun D Chang CJ Mitochondrial-targeted fluorescent probes for reactive oxygen species Curr Opin Chem Biol 2010 14 50 56 19910238 10.1016/j.cbpa.2009.10.014 PMC2830890

[B110] Koide Y Urano Y Kenmoku S Kojima H Nagano T Design and synthesis of fluorescent probes for selective detection of highly reactive oxygen species in mitochondria of living cells J Am Chem Soc 2007 129 10324 10325 17672465 10.1021/ja073220m

[B111] Nishikawa T Edelstein D Du XL Yamagishi S Matsumura T Kaneda Y Yorek MA Beebe D Oates PJ Hammes HP Normalizing mitochondrial superoxide production blocks three pathways of hyperglycaemic damage Nature 2000 404 787 790 10783895 10.1038/35008121

[B112] Srinivasan S Yeh M Danziger EC Hatley ME Riggan AE Leitinger N Berliner JA Hedrick CC Glucose regulates monocyte adhesion through endothelial production of interleukin-8 Circ Res 2003 92 371 377 12600878 10.1161/01.RES.0000061714.74668.5C

[B113] Pearlstein DP Ali MH Mungai PT Hynes KL Gewertz BL Schumacker PT Role of mitochondrial oxidant generation in endothelial cell responses to hypoxia Arterioscler Thromb Vasc Biol 2002 22 566 573 11950692 10.1161/01.atv.0000012262.76205.6a

[B114] Schafer M Schafer C Ewald N Piper HM Noll T Role of redox signaling in the autonomous proliferative response of endothelial cells to hypoxia Circ Res 2003 92 1010 1015 12690038 10.1161/01.RES.0000070882.81508.FC

[B115] Moreira ES Brasch NE Yun J Vitamin B12 protects against superoxide-induced cell injury in human aortic endothelial cells Free Radic Biol Med 2011 51 876 883 21672628 10.1016/j.freeradbiomed.2011.05.034 PMC3163124

[B116] Smith RA Porteous CM Gane AM Murphy MP Delivery of bioactive molecules to mitochondria in vivo Proc Natl Acad Sci U S A 2003 100 5407 5412 12697897 10.1073/pnas.0931245100 PMC154358

[B117] Rodriguez-Cuenca S Cocheme HM Logan A Abakumova I Prime TA Rose C Vidal-Puig A Smith AC Rubinsztein DC Fearnley IM Consequences of long-term oral administration of the mitochondria-targeted antioxidant MitoQ to wild-type mice Free Radic Biol Med 2010 48 161 172 19854266 10.1016/j.freeradbiomed.2009.10.039

[B118] Graham D Huynh NN Hamilton CA Beattie E Smith RA Cocheme HM Murphy MP Dominiczak AF Mitochondria-targeted antioxidant MitoQ10 improves endothelial function and attenuates cardiac hypertrophy Hypertension 2009 54 322 328 19581509 10.1161/HYPERTENSIONAHA.109.130351

[B119] Adlam VJ Harrison JC Porteous CM James AM Smith RA Murphy MP Sammut IA Targeting an antioxidant to mitochondria decreases cardiac ischemia-reperfusion injury Faseb J 2005 19 1088 1095 15985532 10.1096/fj.05-3718com

[B120] Chacko BK Reily C Srivastava A Johnson MS Ye Y Ulasova E Agarwal A Zinn KR Murphy MP Kalyanaraman B Darley-Usmar V Prevention of diabetic nephropathy in Ins2(+/)(−)(AkitaJ) mice by the mitochondria-targeted therapy MitoQ Biochem J 2010 432 9 19 20825366 10.1042/BJ20100308 PMC2973231

[B121] Lowes DA Thottakam BM Webster NR Murphy MP Galley HF The mitochondria-targeted antioxidant MitoQ protects against organ damage in a lipopolysaccharide-peptidoglycan model of sepsis Free Radic Biol Med 2008 45 1559 1565 18845241 10.1016/j.freeradbiomed.2008.09.003

[B122] Supinski GS Murphy MP Callahan LA MitoQ administration prevents endotoxin-induced cardiac dysfunction Am J Physiol Regul Integr Comp Physiol 2009 297 R1095 R1102 19657095 10.1152/ajpregu.90902.2008 PMC2763820

[B123] Esplugues JV Rocha M Nunez C Bosca I Ibiza S Herance JR Ortega A Serrador JM D'Ocon P Victor VM Complex I dysfunction and tolerance to nitroglycerin: an approach based on mitochondrial-targeted antioxidants Circ Res 2006 99 1067 1075 17053193 10.1161/01.RES.0000250430.62775.99

[B124] Kuznetsov AV Kehrer I Kozlov AV Haller M Redl H Hermann M Grimm M Troppmair J Mitochondrial ROS production under cellular stress: comparison of different detection methods Anal Bioanal Chem 2011 400 2383 2390 21336935 10.1007/s00216-011-4764-2

[B125] Hardy M Chalier F Ouari O Finet JP Rockenbauer A Kalyanaraman B Tordo P Mito-DEPMPO synthesized from a novel NH2-reactive DEPMPO spin trap: a new and improved trap for the detection of superoxide Chem Commun (Camb) 2007 1083 1085 17325813 10.1039/b616076j

[B126] Quintero M Colombo SL Godfrey A Moncada S Mitochondria as signaling organelles in the vascular endothelium Proc Natl Acad Sci U S A 2006 103 5379 5384 16565215 10.1073/pnas.0601026103 PMC1459363

[B127] Blouin A Bolender RP Weibel ER Distribution of organelles and membranes between hepatocytes and nonhepatocytes in the rat liver parenchyma. A stereological study J Cell Biol 1977 72 441 455 833203 10.1083/jcb.72.2.441 PMC2110997

[B128] Dranka BP Hill BG Darley-Usmar VM Mitochondrial reserve capacity in endothelial cells: The impact of nitric oxide and reactive oxygen species Free Radic Biol Med 2010 48 905 914 20093177 10.1016/j.freeradbiomed.2010.01.015 PMC2860730

[B129] Widlansky ME Gutterman DD Regulation of endothelial function by mitochondrial reactive oxygen species Antioxid Redox Signal 2011 15 1517 1530 21194353 10.1089/ars.2010.3642 PMC3151425

[B130] Wang Y Zang QS Liu Z Wu Q Maass D Dulan G Shaul PW Melito L Frantz DE Kilgore JA Regulation of VEGF-induced endothelial cell migration by mitochondrial reactive oxygen species Am J Physiol Cell Physiol 2011 301 C695 C704 21653897 10.1152/ajpcell.00322.2010 PMC3174570

[B131] Liu Y Zhao H Li H Kalyanaraman B Nicolosi AC Gutterman DD Mitochondrial sources of H2O2 generation play a key role in flow-mediated dilation in human coronary resistance arteries Circ Res 2003 93 573 580 12919951 10.1161/01.RES.0000091261.19387.AE

[B132] Weber C Noels H Atherosclerosis: current pathogenesis and therapeutic options Nat Med 2011 17 1410 1422 22064431 10.1038/nm.2538

[B133] Roy Chowdhury SK Sangle GV Xie X Stelmack GL Halayko AJ Shen GX Effects of extensively oxidized low-density lipoprotein on mitochondrial function and reactive oxygen species in porcine aortic endothelial cells Am J Physiol Endocrinol Metab 2010 298 E89 E98 19843872 10.1152/ajpendo.00433.2009

[B134] Takabe W Li R Ai L Yu F Berliner JA Hsiai TK Oxidized low-density lipoprotein-activated c-Jun NH2-terminal kinase regulates manganese superoxide dismutase ubiquitination: implication for mitochondrial redox status and apoptosis Arterioscler Thromb Vasc Biol 2010 30 436 441 20139358 10.1161/ATVBAHA.109.202135 PMC3324126

[B135] Libby P Ridker PM Hansson GK Progress and challenges in translating the biology of atherosclerosis Nature 2011 473 317 325 21593864 10.1038/nature10146

[B136] Matsumoto T Kobayashi T Kamata K Role of lysophosphatidylcholine (LPC) in atherosclerosis Curr Med Chem 2007 14 3209 3220 18220755 10.2174/092986707782793899

[B137] Haffner SJ Cassells H Hyperglycemia as a cardiovascular risk factor Am J Med 2003 115 Suppl 8A 6S 11S 14678859 10.1016/j.amjmed.2003.09.009

[B138] Suzuki K Olah G Modis K Coletta C Kulp G Gero D Szoleczky P Chang T Zhou Z Wu L Hydrogen sulfide replacement therapy protects the vascular endothelium in hyperglycemia by preserving mitochondrial function Proc Natl Acad Sci U S A 2011 108 13829 13834 21808008 10.1073/pnas.1105121108 PMC3158211

[B139] Ungvari Z Labinskyy N Mukhopadhyay P Pinto JT Bagi Z Ballabh P Zhang C Pacher P Csiszar A Resveratrol attenuates mitochondrial oxidative stress in coronary arterial endothelial cells Am J Physiol Heart Circ Physiol 2009 297 H1876 H1881 19749157 10.1152/ajpheart.00375.2009 PMC2781360

[B140] Rajesh M Mukhopadhyay P Batkai S Hasko G Liaudet L Drel VR Obrosova IG Pacher P Cannabidiol attenuates high glucose-induced endothelial cell inflammatory response and barrier disruption Am J Physiol Heart Circ Physiol 2007 293 H610 H619 17384130 10.1152/ajpheart.00236.2007 PMC2228254

[B141] Zheng Z Chen H Wang H Ke B Zheng B Li Q Li P Su L Gu Q Xu X Improvement of retinal vascular injury in diabetic rats by statins is associated with the inhibition of mitochondrial reactive oxygen species pathway mediated by peroxisome proliferator-activated receptor gamma coactivator 1alpha Diabetes 2010 59 2315 2325 20566666 10.2337/db10-0638 PMC2927955

[B142] Wang J Alexanian A Ying R Kizhakekuttu TJ Dharmashankar K Vasquez-Vivar J Gutterman DD Widlansky ME Acute Exposure to Low Glucose Rapidly Induces Endothelial Dysfunction and Mitochondrial Oxidative Stress: Role for AMP Kinase Arterioscler Thromb Vasc Biol 2012 32 712 720 22207730 10.1161/ATVBAHA.111.227389 PMC3319449

[B143] Arruda RM Peotta VA Meyrelles SS Vasquez EC Evaluation of vascular function in apolipoprotein E knockout mice with angiotensin-dependent renovascular hypertension Hypertension 2005 46 932 936 16087779 10.1161/01.HYP.0000182154.61862.52

[B144] Murray PJ Wynn TA Protective and pathogenic functions of macrophage subsets Nat Rev Immunol 2011 11 723 737 21997792 10.1038/nri3073 PMC3422549

[B145] West AP Brodsky IE Rahner C Woo DK Erdjument-Bromage H Tempst P Walsh MC Choi Y Shadel GS Ghosh S TLR signalling augments macrophage bactericidal activity through mitochondrial ROS Nature 2011 472 476 480 21525932 10.1038/nature09973 PMC3460538

[B146] Del Prete A Zaccagnino P Di Paola M Saltarella M Oliveros Celis C Nico B Santoro G Lorusso M **Role of mitochondria and reactive oxygen species in dendritic cell differentiation and functions** Free Radic Biol Med 2008 44 1443 1451 18242195 10.1016/j.freeradbiomed.2007.12.037

[B147] Gerthoffer WT Mechanisms of vascular smooth muscle cell migration Circ Res 2007 100 607 621 17363707 10.1161/01.RES.0000258492.96097.47

[B148] Lee SJ Seo KW Yun MR Bae SS Lee WS Hong KW Kim CD 4-Hydroxynonenal enhances MMP-2 production in vascular smooth muscle cells via mitochondrial ROS-mediated activation of the Akt/NF-kappaB signaling pathways Free Radic Biol Med 2008 45 1487 1492 18805481 10.1016/j.freeradbiomed.2008.08.022

[B149] Bailey SR Mitra S Flavahan S Flavahan NA Reactive oxygen species from smooth muscle mitochondria initiate cold-induced constriction of cutaneous arteries Am J Physiol Heart Circ Physiol 2005 289 H243 H250 15764673 10.1152/ajpheart.01305.2004

[B150] Sato H Sato M Kanai H Uchiyama T Iso T Ohyama Y Sakamoto H Tamura J Nagai R Kurabayashi M Mitochondrial reactive oxygen species and c-Src play a critical role in hypoxic response in vascular smooth muscle cells Cardiovasc Res 2005 67 714 722 15913578 10.1016/j.cardiores.2005.04.017

[B151] Vander Heiden MG Cantley LC Thompson CB Understanding the Warburg effect: the metabolic requirements of cell proliferation Science 2009 324 1029 1033 19460998 10.1126/science.1160809 PMC2849637

[B152] Koppenol WH Bounds PL Dang CV Otto Warburg's contributions to current concepts of cancer metabolism Nat Rev Cancer 2011 11 325 337 21508971 10.1038/nrc3038

[B153] Wellen KE Thompson CB Cellular metabolic stress: considering how cells respond to nutrient excess Mol Cell 2010 40 323 332 20965425 10.1016/j.molcel.2010.10.004 PMC3190402

[B154] Christofk HR Vander Heiden MG Harris MH Ramanathan A Gerszten RE Wei R Fleming MD Schreiber SL Cantley LC **The M2 splice isoform of pyruvate kinase is important for cancer metabolism and tumour growth** Nature 2008 452 230 233 18337823 10.1038/nature06734

[B155] Anastasiou D Poulogiannis G Asara JM Boxer MB Jiang JK Shen M Bellinger G Sasaki AT Locasale JW Auld DS Inhibition of pyruvate kinase M2 by reactive oxygen species contributes to cellular antioxidant responses Science 2011 334 1278 1283 22052977 10.1126/science.1211485 PMC3471535

[B156] Hamanaka RB Chandel NS Cell biology. Warburg effect and redox balance Science 2011 334 1219 1220 22144609 10.1126/science.1215637

[B157] Harrison DG Gongora MC Oxidative stress and hypertension Med Clin North Am 2009 93 621 635 19427495 10.1016/j.mcna.2009.02.015

[B158] Lavoie JL Sigmund CD Minireview: overview of the renin-angiotensin system–an endocrine and paracrine system Endocrinology 2003 144 2179 2183 12746271 10.1210/en.2003-0150

[B159] Widder JD Fraccarollo D Galuppo P Hansen JM Jones DP Ertl G Bauersachs J Attenuation of angiotensin II-induced vascular dysfunction and hypertension by overexpression of Thioredoxin 2 Hypertension 2009 54 338 344 19506101 10.1161/HYPERTENSIONAHA.108.127928 PMC2752391

[B160] Ballinger SW Patterson C Knight-Lozano CA Burow DL Conklin CA Hu Z Reuf J Horaist C Lebovitz R Hunter GC Mitochondrial integrity and function in atherogenesis Circulation 2002 106 544 549 12147534 10.1161/01.cir.0000023921.93743.89

[B161] Fujimoto H Taguchi J Imai Y Ayabe S Hashimoto H Kobayashi H Ogasawara K Aizawa T Yamakado M Nagai R Ohno M Manganese superoxide dismutase polymorphism affects the oxidized low-density lipoprotein-induced apoptosis of macrophages and coronary artery disease Eur Heart J 2008 29 1267 1274 17967822 10.1093/eurheartj/ehm500

[B162] Ciccone S Maiani E Bellusci G Diederich M Gonfloni S Parkinson’S disease: a complex interplay of mitochondrial DNA alterations and oxidative stress Int J Mol Sci 2013 14 2388 2409 23348931 10.3390/ijms14022388 PMC3587993

[B163] Xie H Lev D Gong Y Wang S Pollock RE Wu X Gu J Reduced mitochondrial DNA copy number in peripheral blood leukocytes increases the risk of soft tissue sarcoma Carcinogenesis 2013 10.1093/carcin/bgt023 23349016

[B164] Krzywanski DM Moellering DR Fetterman JL Dunham-Snary KJ Sammy MJ Ballinger SW The mitochondrial paradigm for cardiovascular disease susceptibility and cellular function: a complementary concept to Mendelian genetics Lab Invest 2011 91 1122 1135 21647091 10.1038/labinvest.2011.95 PMC3654682

[B165] Gilgun-Sherki Y Melamed E Offen D The role of oxidative stress in the pathogenesis of multiple sclerosis: the need for effective antioxidant therapy J Neurol 2004 251 261 268 15015004 10.1007/s00415-004-0348-9

[B166] Filippin LI Vercelino R Marroni NP Xavier RM Redox signalling and the inflammatory response in rheumatoid arthritis Clin Exp Immunol 2008 152 415 422 18422737 10.1111/j.1365-2249.2008.03634.x PMC2453196

[B167] Burek CL Rose NR Autoimmune thyroiditis and ROS Autoimmun Rev 2008 7 530 537 18625441 10.1016/j.autrev.2008.04.006

[B168] Chen J Gusdon AM Thayer TC Mathews CE Role of increased ROS dissipation in prevention of T1D Ann N Y Acad Sci 2008 1150 157 166 19120287 10.1196/annals.1447.045 PMC3817822

[B169] Simon A Park H Maddipati R Lobito AA Bulua AC Jackson AJ Chae JJ Ettinger R de Koning HD Cruz AC Concerted action of wild-type and mutant TNF receptors enhances inflammation in TNF receptor 1-associated periodic fever syndrome Proc Natl Acad Sci U S A 2010 107 9801 9806 20457915 10.1073/pnas.0914118107 PMC2906866

[B170] Vivekananthan DP Penn MS Sapp SK Hsu A Topol EJ Use of antioxidant vitamins for the prevention of cardiovascular disease: meta-analysis of randomised trials Lancet 2003 361 2017 2023 12814711 10.1016/S0140-6736(03)13637-9

[B171] Bjelakovic G Nikolova D Gluud LL Simonetti RG Gluud C Mortality in randomized trials of antioxidant supplements for primary and secondary prevention: systematic review and meta-analysis Jama 2007 297 842 857 17327526 10.1001/jama.297.8.842

[B172] Sesso HD Buring JE Christen WG Kurth T Belanger C MacFadyen J Bubes V Manson JE Glynn RJ Gaziano JM Vitamins E and C in the prevention of cardiovascular disease in men: the Physicians' Health Study II randomized controlled trial Jama 2008 300 2123 2133 18997197 10.1001/jama.2008.600 PMC2586922

[B173] Cook NR Albert CM Gaziano JM Zaharris E MacFadyen J Danielson E Buring JE Manson JE A randomized factorial trial of vitamins C and E and beta carotene in the secondary prevention of cardiovascular events in women: results from the Women's Antioxidant Cardiovascular Study Arch Intern Med 2007 167 1610 1618 17698683 10.1001/archinte.167.15.1610 PMC2034519

